# Estimated Impact of Achieving the Australian National Sodium Reduction Targets on Blood Pressure, Chronic Kidney Disease Burden and Healthcare Costs: A Modelling Study

**DOI:** 10.3390/nu15020318

**Published:** 2023-01-09

**Authors:** Leopold Ndemnge Aminde, Mary Njeri Wanjau, Linda J. Cobiac, J. Lennert Veerman

**Affiliations:** Public Health & Economics Modelling Group, School of Medicine & Dentistry, Griffith University, Gold Coast, QLD 4222, Australia

**Keywords:** primary prevention, salt, incidence, mortality, kidney disease, health expenditure

## Abstract

Excess sodium intake raises blood pressure which increases the risk of chronic kidney disease (CKD). We aimed to estimate the impact of reduced sodium intake on future CKD burden in Australia. A multi-cohort proportional multistate lifetable model was developed to estimate the potential impact on CKD burden and health expenditure if the Australian Suggested Dietary Target (SDT) and the National Preventive Health Strategy 2021–2030 (NPHS) sodium target were achieved. Outcomes were projected to 2030 and over the lifetime of adults alive in 2019. Achieving the SDT and NPHS targets could lower population mean systolic blood pressure by 2.1 mmHg and 1.7 mmHg, respectively. Compared to normal routines, attaining the SDT and NPHS target by 2030 could prevent 59,220 (95% UI, 53,140–65,500) and 49,890 (44,377–55,569) incident CKD events, respectively, while postponing 568 (479–652) and 511 (426–590) CKD deaths, respectively. Over the lifetime, this generated 199,488 health-adjusted life years (HALYs) and AUD 644 million in CKD healthcare savings for the SDT and 170,425 HALYs and AUD 514 million for the NPHS. CKD due to hypertension and CKD due to other/unspecified causes were the principal contributors to the HALY gains. Lowering sodium consumption in Australia could deliver substantial CKD health and economic benefits.

## 1. Introduction

Excess sodium intake is a leading dietary health risk. In 2017, high sodium diets were responsible for more than 3 million deaths and over 70 million disability-adjusted life years (DALYs) worldwide [1]. One major way through which excess sodium intake affects health is by raising blood pressure (BP). The INTERSALT study that included over 10,000 adults from 32 countries was the earliest large-scale epidemiological study that demonstrated that high urinary sodium excretion (a proxy for intake) and a high sodium-potassium ratio assessed via 24 h urine were associated with increases in BP [2]. Meta-analyses of randomized trials have confirmed a robust linear and dose–response relationship between sodium intake and BP. This causal effect is stronger in those with hypertension, Black ethnic populations and greater for systolic BP than diastolic BP [3,4].

In Australia, Huggins and colleagues were the first to demonstrate this link between sodium and BP. Using data from the Melbourne Collaborative Cohort Study, they found that after adjusting for age, body mass index, country of birth and anti-hypertensive medication use, a 100 mmol/day increase in sodium was associated with a 2.3 mmHg increase in systolic BP [5]. Furthermore, the Australian Burden of Disease 2015 study revealed that 21% of the burden of hypertension in Australia was due to diets high in sodium [6].

Lowering sodium intake in the population has been shown to reduce average BP and reduces the risk of developing vascular events, such as CKD and the advancement of kidney injury to End Stage Kidney Disease [7,8,9]. A recent Cochrane meta-analysis of randomized trials in people with CKD demonstrated that a reduction in salt intake by 4.2 g (1690 mg sodium) per day lowered systolic and diastolic BP by 6.91 (4.99–8.82) mmHg and 3.91 (3.02–4.80) mmHg, respectively. In addition, for people with early stages of CKD, this salt reduction led to a 36% (26–44) reduction in albuminuria, a potent marker of poor kidney function [10]. Thus, in addition to reducing the risk of incident CKD, decreasing average population sodium consumption could play a significant role in reducing the morbidity and mortality associated with CKD in Australia.

In 2019, an estimated 62% of the CKD deaths and 56% of CKD-related DALYs lost in Australia were due to hypertension [11]. Despite compelling evidence on the relationship between dietary sodium, BP and CKD, few studies have assessed the long-term impact of sodium reduction on CKD burden [12]. Most modelling studies globally [13,14,15,16,17] and in Australia [18,19] have either focused on the impact on heart disease and stroke or did not explore the longer-term impacts on CKD [20]. This implies the currently reported health impacts of sodium reduction in Australia are likely underestimated. With Australians currently consuming nearly twice the recommended levels of sodium [6], the aim of this study was to complement the above-mentioned knowledge gap by developing a model to quantify the avoidable burden of CKD and savings in health expenditure that could be realised if Australians lowered their average sodium intake to healthier levels.

## 2. Materials and Methods

### 2.1. Specification of the Modelled Sodium Targets

Two main sodium policy targets were modelled in this study: the Australian Suggested Dietary Target (SDT) and the National Preventive Health Strategy (NPHS) 2021–2030 target [21,22]. The National Health and Medical Research Council developed nutrient reference values (NRV) for a range of nutrients including sodium in 2006 and updated these in 2017 to reflect best contemporary evidence. As a result, the SDT, defined as *a daily average intake from food and beverages for certain nutrients that may help in prevention of chronic disease* was established for sodium as 2000 mg (5 g of salt) per day [21]. The recent Australian National Preventive Health Strategy (NPHS) 2021–2030 specifies a long-term approach to prevention and identifies key focus areas for the next 10 years. With respect to healthy diets, a key target in the NPHS is to reduce sodium intakes by an average of 30% by 2030 [22]. In this analysis, we compared the impact of current population sodium intake with a counterfactual (attaining the policy targets) on future CKD burden, assuming a steady linear reduction over ten years towards the respective targets.

### 2.2. Sodium Intake and Blood Pressure Data

Data on sodium intake was obtained from a meta-analysis of 31 published studies and 1 unpublished dataset of surveys conducted across Australia between 1989 to 2015 [23]. After adjusting for non-urinary excretion, the overall weighted average daily intake was ~3700 mg of sodium (9.6 g of salt) based on 24 h urine collections. This meta-analysis included results from the 2011–2012 National Nutrition and Physical Activity Survey (NNPAS) [24], a nationally representative survey of over 12,000 adult Australians that estimated sodium intake using 24 h dietary recall. We used the weighted average 24 h urine sodium estimates from the meta-analysis as baseline sodium intake levels in our modelling. Age- and sex-specific patterns in sodium intake were derived from the NNPAS to adjust the overall estimates accordingly. This was done to account for the age-specific differences in baseline sodium intake as we modelled the relationship with blood pressure through multiple age-cohorts.

Data on blood pressure (BP) was taken from the Australian 2017–2018 National Health Survey [25]. This nationally representative survey used a random multistage area sampling of private dwellings, with a final sample of 16,384 dwellings and 21,315 participants. It was designed to collect data on chronic health conditions and risk factors, such as BP, obesity, smoking, alcohol consumption and physical activity. Consenting adults (18 years and above) typically had two BP measurements taken and the second reading was used. When a third reading was obtained, the average of the second and third readings was recorded as the measured BP, unless the third reading differed by more than 20 mmHg. Measurement was considered invalid if all three BP readings differed by 20 mmHg or more [26]. Estimates of the weighted mean measured BP by age-groups and sex were used for our analysis. See Supplemental Material for all model input data.

### 2.3. Model Framework

We developed a proportional multistate lifetable (PMSLT) Markov model, a dynamic epidemiological model suited for simulating long-term population impacts of interventions for chronic disease prevention [27]. This analysis took a three-step approach: first, the effect of a changes in sodium on BP; second, the effect of changes in blood pressure on incident CKD; and finally, the effect of changes in CKD morbidity and mortality on health-adjusted life years (HALY) and health expenditure. All simulations are conducted for two identical Australian populations, except that one receives the modelled intervention (sodium reduction with changes in blood pressure) and the other continues with business as usual. The difference in health and economic outcomes between the two populations quantifies the impact of the intervention.

#### 2.3.1. Sodium and Blood Pressure Risk Modelling

The change in systolic BP from a change in sodium consumption was based on a Cochrane meta-analysis of randomized trials of interventions conducted over at least a month, as these are considered relevant from a public health perspective [28]. This meta-analysis demonstrated that salt reduction by 4.4 grams (~1720 mg sodium) led to a 5.39 (95% CI 4.15 to 6.62) mmHg and 2.42 (1.29 to 3.56) mmHg drop in systolic BP for people with and without hypertension, respectively. We accounted for this differential impact of changes in sodium for people with mean systolic BP above and below 140 mmHg.

Data on the relative risks of CKD morbidity or mortality due to changes in systolic BP and baseline data on the epidemiology of CKD in Australia were obtained from the 2019 Global Burden of Disease (GBD) study [11]. Blood pressure was modelled as a continuous variable assuming a normal distribution and we assumed stable trends into the future. We calculated the impact of the resulting change in SBP on CKD using the ‘distribution shift’ method of the potential impact fraction (PIF) [29]. In the PIF calculation, we assume a theoretical minimum risk exposure level for systolic BP of 115 mmHg based on evidence from individual participant data meta-analyses of prospective cohort studies on elevated BP and the risk of vascular events [30]. By integrating the risk function with the exposure (systolic BP) distribution for each five year age cohort by sex, with and without the intervention (sodium reduction), the PIF quantified the proportional change in the incidence of CKD (See Supplemental Material).

#### 2.3.2. Epidemiological and Multistate Lifetable Modelling

We modelled the different CKD causes (CKD due to hypertension, CKD due to diabetes mellitus, CKD due to glomerulonephritis and CKD due to other and unspecified causes) as implemented in the GBD study [31]. CKD was defined in the GBD as elevated urinary albumin to creatinine ratio, decreased estimated glomerular filtration rate, or end-stage kidney disease. Each CKD cause was modelled as a separate disease Markov sub-model in the PMSLT, in which, for each cause, proportions of the population move between four Markov health states: Healthy (Alive without CKD), Diseased (that is, Alive with CKD/prevalent CKD), Dead from CKD (that is, CKD-specific deaths which is a function of case fatality) and Dead from other (non-CKD) causes, in 1-year cycles. State transitions are informed by incidence, remission and case fatality hazards, with death an absorbing state. The DISMOD-II epidemiological software was used to derive CKD case fatality rates for each of the CKD causes by sex and age-group cohort, which are seldom available. This analytical software uses a set of differential equations that exploit the causal relation in a typical disease process to estimate scarce epidemiological parameters (such as case fatality rates) while maintaining stability in the overall disease epidemiology [32]. Given the progressive nature of CKD, we assumed zero remission. These disease progression Markov models were linked to the lifetable. The PIF described above calculates new (post-intervention) incidence rates of CKD, which over time, leads to changes in prevalence, with mortality being a function of prevalence. This disease-specific mortality propagates into the lifetable which recalculates all-cause mortality and life years. Life years are adjusted for loss of quality life due to CKD for each age and sex to obtain health-adjusted life years (HALY). The poor quality of life adjustment was estimated by dividing age and sex-specific years lived with disability (YLD) for each CKD cause by the corresponding prevalence to obtain the disease-specific disability weight. We corrected this for disability from background health conditions using age- and sex-specific all-cause YLD from the GBD [11]. One HALY could thus be defined as a year of life in perfect health. Sex and five-year age-group cohort simulations were conducted in annual cycles over the lifetime (till people died or reached 100 years of age) of a closed cohort of adult Australians alive in 2019, simultaneously for the business as usual and intervention populations. The health outcomes quantified included absolute and relative changes in CKD incidence and mortality and the HALYs.

#### 2.3.3. Healthcare Costs

Cost data for CKD were obtained from the Australian Institute of Health and Welfare (AIHW) estimates for health expenditure 2018–2019 [33]. The AIHW pools data from multiple hospital, morbidity, non-admitted patient, and pharmaceutical datasets covering public, private and primary care services including general practitioner and allied health services, pathology and medical imaging, patient admissions, emergency and outpatient services, out-of-hospital services and prescription pharmaceuticals from across the country. It maps these to specific areas of expenditure and to disease conditions using ICD-10-AM codes while implementing a predominantly top-down costing approach. Further details are reported elsewhere [34]. We divided the total annual health spending for CKD by the corresponding five year age group and sex-specific prevalent CKD cases to obtain the annual per capita costs for each of the CKD causes. The resulting estimates were multiplied by the projected changes in prevalent CKD types due the policy targets to estimate the impacts on CKD health expenditure. Our model base year was 2019 so we did not need inflation adjustments. Costs projections beyond the base year were discounted [35].

### 2.4. Scenario and Uncertainty Analysis

Our base case analyses included: (i) reducing current sodium intake to an average of 2000 mg of sodium or 5 g of salt (Australian SDT) and (ii) Reducing average sodium intake by 30% (NPHS, 2021–2030). In addition, we conducted the following scenario analyses: (iii) a 20% relative reduction in sodium intake, (iv) a 15% relative reduction in sodium intake (similar to that achieved in the UK salt reduction program), (v) a reduction in salt intake by 1 g, and (vi) a reduction in salt intake by 2 g. In the base case and scenario analyses, we present undiscounted HALYs while costs are discounted at 3%. We did further sensitivity analyses with HALYs discounted at 3% and costs discounted at 5% [35].

We conducted probabilistic sensitivity analysis to account for the impact of uncertainty in model input parameters on the outcomes. Plausible statistical distributions for parameters with known uncertainty were specified: sodium intake (normal), blood pressure (normal), relative risks (lognormal), sodium-BP relationship (normal), costs (gamma), from which random draws were made using Monte Carlo simulations with bootstrapping implemented with the Ersatz software version 1.35 [36]. We ran 2000 simulations and report the 95% uncertainty intervals (2.5th and 97.5th percentiles).

The study methods and interpretation of results adheres to the Guidelines for Accurate and Transparent Health Estimates Reporting (GATHER) statement [37]. Ethics approval was deemed not applicable for this analysis given that it was a modelling study that did not involve any interaction with human participants and mainly used publicly available de-identified data.

## 3. Results

### 3.1. Impact of Sodium Reduction on Mean Blood Pressure

To achieve the Australian SDT by 2030, our model estimates that a modest linear and sustained reduction from current sodium levels, that is, on average 0.46 g of salt (~180 mg sodium) annually for 11 years would be required. This could lower mean systolic BP by 2.93 mmHg in men and 1.3 mmHg in women. Second, achieving the NPHS target by 2030 would require a sustained average annual decline of 0.29 g of salt (~110 mg sodium). This could reduce mean systolic BP in 2030 by 1.91 mmHg and 1.42 mmHg for men and women, respectively. Table 1 depicts systolic BP distributions with and without the intervention.

### 3.2. Estimated Changes in CKD Incidence by 2030 and over the Lifetime

Between 2019 and 2030, attaining the SDT could prevent over 59,220 (95% uncertainty intervals [UI]: 53,140 to 65,500) new cases of CKD overall. In the same period, achieving the NPHS target could prevent 49,890 (95% UI: 44,370 to 55,570) new cases of CKD. If the sodium targets were sustained for the remaining lifetime, this would result in 386,800 (95% UI: 347,450 to 427,680) and 348,325 (312,980 to 386,620) incident cases of CKD avoided for the SDT and NPHS target, respectively. For both targets, most of the number of incident cases avoided over the lifetime were CKD of unspecified or other causes (53%) followed by CKD due to hypertension (23%). Furthermore, relative reductions in cumulative incident CKD over the lifetime were bigger in men compared to women, that is, 8.3% vs. 3.2% for SDT and 6.1% vs. 3.8% for NPHS. See Table 2 and Supplemental Material, Figures S3–S6.

### 3.3. Estimated Changes in CKD Mortality by 2030 and Lifetime

Over 11 years, achieving the Australian SDT could prevent 570 (95% UI: 479 to 652) deaths from CKD while reaching the NPHS target by 2030 could prevent 511 (95% UI: 426 to 590) CKD deaths. Over the lifetime, over 22,500 (20,400 to 24,750) and 20,290 (18,440 to 22,275) deaths for SDT and NPHS targets, respectively, could be averted. In the two targets, CKD due to hypertension (40%) and CKD due to glomerulonephritis (30%), had the largest deaths averted. Estimated relative reductions in cumulative CKD mortality over the lifetime of women were 1.7% for the SDT and 2.2% for the NPHS, while over the lifetime of men, reductions were 4.0% for the SDT and 2.9% for the NPHS. See Table 3 and Supplemental Material, Figures S3, S4, S7 and S8.

### 3.4. Healthy Life Years Gained between 2019–2030 and Lifetime

**Figure 1:**
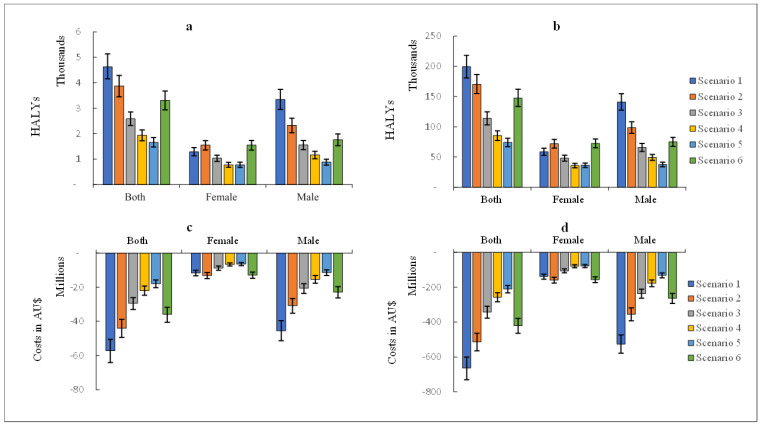
Health-adjusted life years (HALY) gained and CKD-related health expenditure savings. Panel (**a**) depicts the HALYs gained between 2019–2030 and panel (**b**) shows HALYs gained over the lifetime of Australians alive in 2019 by gender and for each of the six scenarios modelled; Panel (**c**) shows the CKD health expenditure savings between 2019–2030 while panel (**d**) shows the CKD health expenditure savings over the remaining lifetime of Australians alive in 2019. AUD, 2019 Australian dollars. Error bars reflect the 95% uncertainty intervals. Scenario 1: Reducing current salt intake to an average of 5 g/day (Australian Suggested Dietary Target); Scenario 2: a 30% relative reduction in current salt intake (National Preventive Health Strategy, 2021–2030 target); Scenario 3: 20% relative reduction in current salt intake; Scenario 4: 15% relative reduction in current salt intake; Scenario 5: reduction in current salt intake by 1 g; Scenario 6: reduction in current salt intake by 2 g. HALY, health-adjusted life years.

Achieving the SDT and NPHS targets were estimated to result in 4645 CKD-related HALYs (that is, 26.7 HALYs per 100,000 persons) and 3870 CKD-related HALYs (that is, 22.3 HALYs per 100,000 persons) gained, respectively by 2030. While over the remaining lifetime, if these sodium reduction targets were sustained, we project that about 200,000 HALYs could be gained for the SDT and over 170,000 HALYs for the NPHS target. Over the lifetime, the combined avoidable morbidity and mortality from CKD due to hypertension had the largest (34–35%) contribution to the HALYs gained, followed by CKD due to other or unspecified causes (26%). See Table 4 and Figure 1.

### 3.5. Healthcare Costs

For health expenditure, achieving the SDT and NPHS target could save AUD 57 million and AUD 44 million, respectively, in CKD-related health care costs by 2030. Over the lifetime, these savings increased substantially to AUD 664 million (AUD 38.2 per capita) and AUD 514 million (AUD 29.6 per capita), respectively. See Figure 1.

### 3.6. Scenario Analyses

In the main analysis, we present undiscounted HALYs, and costs were discounted at 3%. Applying a 3% discount rate, HALYs dropped by 22.5% in the first 11 years, and were reduced by 67.6% over the lifetime (Supplemental Material Figure S9). When a 5% discount rate was applied to costs, savings in health expenditure declined by 15.8% in the first 11 years and by 43% over the lifetime. Supplemental Material Figures S10 and S11 depict the discounted costs overall, by gender and by CKD cause over both time horizons. We also modelled the impact of other modest reductions in sodium. For example, a 15% sodium reduction (i.e., half of the NPHS target, but similar to the United Kingdom salt reduction program effect) over 11 years, could prevent 24,900 new CKD cases and avert 255 CKD deaths. This translates to 85,320 CKD-related HALYs gained over their remaining lifetime. Furthermore, if only 1 g of salt (~390 mg sodium) was reduced, that is, from 9.6 g of salt (3700 mg of Na) to 8.6 g of salt (3310 mg of Na), there would be 21,400 fewer new cases of CKD and 225 deaths averted by 2030. Over the lifetime, these increase by 7 times for incidence and 19 times for deaths and results in 74,000 CKD-related HALYs gained. See Table 2 and Table 3, and Supplemental Material Figures S3–S8.

## 4. Discussion

This study projected the impact of linear and sustained reductions in sodium intake towards achieving the National Preventive Health Strategy 2021–2030 and the Suggested Dietary Targets on the burden of CKD in Australia. Based on current sodium and BP distributions for people aged 25 years and above, our modelling shows that achieving the NPHS sodium target could result in 49,800 fewer new cases of CKD and 509 deaths averted by 2030. Furthermore, attaining the Australian SDT for sodium could prevent over 59,000 new CKD cases and postpone 570 CKD deaths by 2030 in Australia. If the target sodium levels are sustained for the remaining lifetime, these impacts substantially increase (about 7 times for incidence and 40 times for deaths), translating to 165,000 HALYs (for NPHS) and 200,000 HALYs (for SDT) gained, with considerable savings in health expenditure. As expected, discounting HALYs and higher discount rate on costs had a significant impact on outcomes. These novel findings demonstrate the potential extra benefits of sodium reduction besides its impact on heart disease and stroke.

### 4.1. Comparison with Other Studies

We estimate that achieving the NPHS and SDT for sodium intake could reduce average systolic BP of adult Australians by 1.7 mmHg and 2.1 mmHg, respectively. High certainty evidence from a recent Cochrane meta-analysis showed that salt reduction of 4.2 g/day (1640 mg of sodium) in people with CKD decreased systolic BP by 6.9 mmHg, and reduced albuminuria by 36% [10]. Studies have shown that the effect of high sodium intake on BP are greater in people with CKD compared to the general non-CKD population, due to decreased renal excretion of the excess sodium [8,38]. As a result, reducing sodium intake is a powerful tool to achieve blood pressure control in people with CKD, thus decelerating disease progression. Moreover, impaired kidney function is itself a prognostic factor for poor cardiovascular outcomes [8]. Thus, population sodium reduction efforts that prevent CKD potentially deliver extra benefits via a ripple effect on CVD burden.

Our literature search identified only one study that has modelled the long-term impacts of sodium reduction on CKD burden [12]. In this Dutch study, Hendriksen et al. used baseline data from the PREVEND study to develop a Markov simulation model. Their projections showed that achieving a 6 g/day target of salt led to 290,000 fewer cases of CKD (1.1% reduction) and 470 fewer cases of End Stage Kidney Disease (3.2% reduction) after 20 years. A recent modelling study in Australia found that full compliance with the Australia’s sodium reformulation program could prevent 565 new CKD cases, avert 40 CKD deaths and 527 CKD-related DALYs a year [20]. These findings are lower than our study results and is likely due to the following reasons. First, the magnitude of sodium reduction achieved from the reformulation program was 107 mg/day, which though similar to the annual reduction for the NPHS target (110 mg/day), is lower than the annual sodium reduction from the SDT (180 mg/day) modelled in our study. Second, their study used comparative risk assessment modelling to estimate the disease burden attributable to sodium. This framework lacks the time component and thus estimates were for a single year. In contrast, our dynamic multistate lifetable Markov model allowed for more nuanced time-dependent projections with linear incremental annual reductions over 11 years (through to 2030) and impacts over the life course. The differences in modelling frameworks, intervention effects and time horizon could explain the differences in health outcomes. However, these studies demonstrate the additional benefits of sodium reduction besides its effect on CVDs.

### 4.2. Implications of the Findings

The Commonwealth Government of Australia has endorsed the WHO global sodium reduction recommendation. However, recent evidence suggests that Australia is not on track to achieve this 30% reduction target by 2025 [39]. Our modelled projections demonstrate the potential CKD-related population health gains that could be missed if current dietary sodium intake patterns persist. Moreover, in addition to the avoidable morbidity that could mitigate increasing hospitalizations and demand for renal replacement therapy [40], significant savings in health expenditure could be obtained. These funds could be invested to further strengthen preventive health efforts or other social causes to improve the wellbeing of Australians.

Most of the sodium in the Australian food system is from pre-packaged and ultra-processed foods [41], hence food reformulation is a viable option to reduce salt intake in the population. The Australian Government Healthy Food Partnership has rightfully set sodium reformulation targets for some of these foods [42]; however, these targets are far from comprehensive. Recent modelling indicates that much larger (more than double) health gains could be achieved if the range of products were as extensive as that implemented in the United Kingdom reformulation program [20]. To maximise impact, there is an urgent need to expand the range of products in this program perhaps through adapting the broad benchmarks recently set by the WHO [43] and making reformulation mandatory. Additionally, there should be regular revision to lower sodium targets in a framework to achieve the National Preventive Health Strategy target of a 30% reduction in sodium intake by 2030 and further down to the Australian SDT of 5 g of salt (2000 mg sodium) per day [21,22].

Our study shows that achieving the SDT (akin to the WHO recommendation) could deliver greater health benefits. Government inaction or delays in enforcing strong policies can have substantial health and economic costs. A recent analysis of the United States Food and Drug Administration’s 4.3 year delay (2016 to 2021) in finalizing their short-term sodium reduction targets was estimated to cost over 250,000 lives over 10 years [44]. Federal and State Governments in Australia must therefore work together to enforce robust and timely policies to avoid such losses. Realizing the policy targets modelled here has the potential to achieve even bigger health gains, through averting other health outcomes related to excess sodium intake, such as heart disease, stroke and stomach cancer [45,46,47]. 

### 4.3. Strengths and Limitations

This study had some limitations. First, our projections do not incorporate trends in dietary sodium intake. Temporal changes in exposure are likely to influence our current projections. However, recent meta-regression of salt consumption in Australia did not find significant evidence of a change in sodium intake in the last two decades. As such, our assumption of stable intake seems conservative. Second, evidence suggests a direct impact of sodium on kidney function independent of BP [48]. However, we modelled the effect mediated through BP, for which the evidence is strongest [7]. It is possible that we may have underestimated the impact by not accounting for additional mechanisms by which sodium affects the kidneys. Third, impaired kidney function is a potent prognostic factor for CVD events and poor outcomes, with the latter also mediated by other risk factors, such as plasma renin, aldosterone, cholesterol and triglyceride. Some of these factors are reportedly influenced by low sodium intake [49,50]. While our study focused on the direct effect of sodium on BP and CKD outcomes only, future modelling studies on the long-term impacts of sodium reduction should consider other indirect and broader health outcomes, including those CKD-related CVD events. While this might not substantially change the current overall recommendations for sodium reduction as a best-buy strategy to curb non-communicable diseases, accurate characterization of these pathophysiological mechanisms could refine modelled projections. Fourth, we did not explicitly disaggregate CKD by the different stages. The CKD prevalent pool in our disease progression Markov model included people of all stages and we applied the same case fatality hazard, on average, based on pooled population data from the GBD 2019 study. We recognize this case fatality assumption as a potential limitation of our modelling, given that outcomes are likely to vary by disease stage [51]. However, our aim was to estimate on average, the population-level impact of sodium reduction on CKD burden. Simulating by CKD stages and specific risk factors is best implemented in microsimulation frameworks, which are nonetheless considerably data-demanding and was beyond the scope of this work. Finally, our modelling does not account for socio-economic differences. Prior studies have suggested that preventive health strategies could have an impact on health inequalities [47], hence future work should evaluate the impacts of sodium reduction on health inequalities in Australia. The health impacts could be potentially big in First Nations People given their comparatively high CKD rates [40].

Our study has the following strengths. First, we maximise the use of Australia-specific data. Sodium intake was based on meta-analysis of surveys conducted across Australia including the 2011–2012 National Nutrition and Physical Activity Survey, corrected for potential underestimation in dietary surveys to obtain weighted 24 h urine estimates. We also used measured BP data from the Australian National Health Survey 2017–2018. Use of these nationally representative data sources enhances the contextual relevance of our study. Second, our dynamic proportional multi-state lifetable model explicitly models age- and sex-specific cohorts of the population thus accounting for the varying impacts in sodium intake and BP distributions. In addition, we account for the differential impact of sodium reduction in people with and without hypertension in line with evidence of the stronger effects of sodium on BP in those with hypertension [4,7]. Third, there is limited empirical evidence on the long-term effects of sodium intake on CKD, echoed in a recent Cochrane meta-analysis [10]. Our modelling compliments this evidence gap by projecting beyond intermediate outcomes (blood pressure) to CKD for the first time in Australia.

## 5. Conclusions

This modelling study suggests that sustained reductions in sodium intake towards attaining the Australian National Preventive Health Strategy 2021–2030 and the NHMRC Suggested Dietary Targets could prevent a substantial CKD burden in Australia and lead to savings in health expenditure. Governments need to step-up efforts with robust and coordinated actions to reduce sodium in the Australian food supply to curb the avoidable health and economic losses in the future. Further studies are warranted to evaluate the impacts of these policy targets on health inequality.

## Figures and Tables

**Table 1:** Estimated changes in mean systolic BP if the national sodium reduction targets were achieved by 2030.

	Men			Women		
Age (Years)	Baseline	SDT Achieved	NPHS Achieved	Baseline	SDT Achieved	NPHS Achieved
25–34	120.3 (13.5)	116.6 (12.7)	118.4 (13.1)	108.5 (15.8)	106.7 (15.3)	107.1 (15.4)
35–44	121.3 (11.6)	118.1 (10.9)	119.5 (11.2)	112.5 (15.7)	111.0 (15.4)	111.2 (15.4)
45–54	126.5 (17.9)	123.3 (17.1)	124.7 (17.5)	119.6 (17.0)	118.1 (16.6)	118.3 (16.7)
55–64	132.4 (14.7)	129.9 (14.1)	130.8 (14.3)	126.8 (20.6)	125.6 (20.4)	125.6 (20.3)
65–74	134.9 (21.3)	132.5 (20.7)	133.4 (20.9)	133.6 (17.7)	132.5 (17.5)	132.4 (17.4)
75–84	136.5 (20.9)	134.7 (20.5)	135.1 (20.6)	137.9 (17.0)	137.2 (16.7)	136.8 (16.8)
≥85	140.2 (33.3)	136.2 (32.8)	137.2 (32.9)	140.8 (28.0)	139.2 (27.8)	138.5 (27.7)

Numbers are mean (standard deviation); SDT, Australian Suggested Dietary Target; NPHS, National Preventive Health Strategy 2021–2030 target.

**Table 2:** Estimated reductions in cumulative incident CKD events in Australia between 2019 to 2030, and over the lifetime.

	**Male**		**Female**		**Total**	
**2019–2030**	**Absolute** **Est. (95% UI)**	**Relative** **Est., % (95% UI)**	**Absolute** **Est. (95% UI)**	**Relative** **Est., % (95% UI)**	**Absolute** **Est. (95% UI)**	**Relative** **Est., % (95% UI)**
SDT, 5 g/day salt target achieved	40,518 (35,890–45,264)	7.4 (6.5–8.2)	18,704 (16,399–21,216)	3.2 (2.8–3.6)	59,223 (53,140–65,503)	5.3 (4.7–5.8)
NPHS 30% relative salt reduction	27,748 (24,194–31,258)	5.0 (4.4–5.6)	22,142 (19,494–24,975)	3.8 (3.4–4.3)	49,890 (44,377–55,569)	4.4 (3.9–4.9)
A 20% relative salt reduction	18,496 (16,182–20,817)	3.4 (2.9–3.8)	14,774 (12,962–16,703)	2.6 (2.3–2.9)	33,270 (29,672–36,947)	3.0 (2.6–3.3)
A 15% relative salt reduction	13,846 (11,988–15,597)	2.5 (2.2–2.8)	11,072 (9611–12,491)	1.9 (1.6–2.2)	24,919 (22,141–27,724)	2.2 (1.9–2.5)
A 1 g/day absolute salt reduction	10,401 (8992–11,817)	1.9 (1.6–2.1)	11,018 (9625–12,578)	1.9 (1.7–2.2)	21,419 (18,967–24,069)	1.9 (1.6–2.1)
A 2 g/day absolute salt reduction	20,838 (18,037–23,603)	3.8 (3.3–4.2)	22,050 (19,126–25,018)	3.8 (3.4–4.3)	42,888 (37,943–47,892)	3.8 (3.4–4.2)
	**Male**		**Female**		**Total**	
**Lifetime**	**Absolute** **Est. (95% UI)**	**Relative** **Est., % (95% UI)**	**Absolute** **Est. (95% UI)**	**Relative** **Est., % (95% UI)**	**Absolute** **Est. (95% UI)**	**Relative** **Est., % (95% UI)**
SDT, 5 g/day salt target achieved	266,676 (237,912–295,250)	8.3 (7.5–9.1)	120,133 (106,868–133,853)	3.4 (3.0–3.7)	386,809 (347,452–427,680)	5.7 (5.2–6.2)
NPHS 30% relative salt reduction	192,140 (171,383–214,418)	6.0 (5.5–6.6)	156,186 (138,862–174,500)	4.4 (4.0–4.8)	348,326 (312,986–386,619)	5.2 (4.7–5.6)
A 20% relative salt reduction	128,163 (113,735–142,551)	4.0 (3.6–4.4)	103,853 (92,544–115,616)	2.9 (2.6–3.2)	232,016 (207,515–257,151)	3.4 (3.1–3.7)
A 15% relative salt reduction	96,067 (85,977–106,793)	3.0 (2.7–3.3)	77,820 (69,266–86,774)	2.2 (2.0–2.4)	173,887 (156,162–192,231)	2.6 (2.3–2.8)
A 1 g/day absolute salt reduction	74,919 (66,529–83,386)	2.3 (2.1–2.6)	79,926 (70,776–89,192)	2.2 (2.0–2.5)	154,845 (138,789–171,916)	2.3 (2.1–2.5)
A 2 g/day absolute salt reduction	149,649 (133,203–166,751)	4.7 (4.2–5.1)	160,061 (142,105–178,186)	4.5 (4.0–5.0)	309,711 (276,892–342,894)	4.6 (4.2–5.0)

SDT, Australian Suggested Dietary Target; NPHS, National Preventive Health Strategy, 2021–2030 target; Est., Estimate; UI, uncertainty intervals.

**Table 3:** Estimated reductions in cumulative mortality from CKD in Australia between 2019 to 2030 and over the lifetime.

	**Male**		**Female**		**Total**	
**2019–2030**	**Absolute** **Est. (95% UI)**	**Relative** **Est., % (95% UI)**	**Absolute** **Est. (95% UI)**	**Relative** **Est., % (95% UI)**	**Absolute** **Est. (95% UI)**	**Relative** **Est., % (95% UI)**
SDT, 5 g/day salt target achieved	423 (341–496)	0.7 (0.6–0.8)	145 (118–171)	0.3 (0.2–0.3)	568 (479–652)	0.5 (0.4–0.6)
NPHS 30% relative salt reduction	313 (249–368)	0.5 (0.4–0.6)	198 (159–231)	0.4 (0.3–0.4)	511 (426–590)	0.5 (0.4–0.5)
A 20% relative salt reduction	209 (169–244)	0.3 (0.3–0.4)	132 (107–153)	0.3 (0.2–0.3)	341 (288–390)	0.3 (0.2–0.3)
A 15% relative salt reduction	157 (124–185)	0.3 (0.2–0.3)	99 (79–116)	0.2 (0.1–0.2)	255 (214–293)	0.2 (0.2–0.3)
A 1 g/day absolute salt reduction	124 (98–146)	0.2 (0.1–0.2)	103 (84–120)	0.2 (0.1–0.2)	226 (188–260)	0.2 (0.1–0.2)
A 2 g/day absolute salt reduction	248 (199–292)	0.4 (0.3–0.5)	205 (163–240)	0.4 (0.3–0.5)	453 (381–520)	0.4 (0.3–0.5)
	**Male**		**Female**		**Total**	
**Lifetime**	**Absolute** **Est. (95% UI)**	**Relative** **Est., % (95% UI)**	**Absolute** **Est. (95% UI)**	**Relative** **Est., % (95% UI)**	**Absolute** **Est. (95% UI)**	**Relative** **Est., % (95% UI)**
SDT, 5 g/day salt target achieved	15,798 (14,248–17,362)	4.0 (3.7–4.4)	6785 (6067–7515)	1.7 (1.6–1.9)	22,583 (20,414–24,752)	2.9 (2.6–3.1)
NPHS 30% relative salt reduction	11,425 (10,286–12,627)	3.0 (2.7–3.2)	8866 (7975–9768)	2.2 (2.1–2.4)	20,291 (18,441–22,276)	2.6 (2.4–2.8)
A 20% relative salt reduction	7638 (6836–8455)	2.0 (1.8–2.1)	5913 (5314–6523)	1.5 (1.4–1.6)	13,551 (12,262–14,891)	1.7 (1.6–1.9)
A 15% relative salt reduction	5730 (5125–6347)	1.5 (1.3–1.6)	4437 (3965–4902)	1.1 (1.0–1.2)	10,168 (9189–11,168)	1.3 (1.2–1.4)
A 1 g/day absolute salt reduction	9047 (8176–9913)	1.1 (1.0–1.3)	4565 (4103–5035)	1.2 (1.1–1.3)	4481 (3995–4963)	1.2 (1.1–1.3)
A 2 g/day absolute salt reduction	8924 (7995–9900)	2.3 (2.0–2.5)	9108 (8162–10,077)	2.3 (2.1–2.5)	18,032 (16,295–19,838)	2.3 (2.1–2.5)

SDT, Australian Suggested Dietary Target; NPHS, National Preventive Health Strategy, 2021–2030 target; Est., Estimate; UI, uncertainty intervals.

**Table 4:** Absolute and relative distribution of total HALY gains by CKD cause over different time horizons.

**Scenario/Time Horizon**	**CKD HTN**	**CKD DM**	**CKD GMN**	**CKD Other**
**2019–2030**				
SDT 5 g/day achieved	1671 (36.1%)	1003 (21.7%)	906 (19.6%)	1031 (22.3%)
NPHS 30% reduction	1411 (36.5%)	833 (21.5%)	744 (19.2%)	877 (22.7%)
20% reduction	942 (36.5%)	556 (21.5%)	496 (19.2%)	582 (22.5%)
15% reduction	707 (36.6%)	418 (21.6%)	373 (19.3%)	437 (22.6%)
1 g/day reduction	607 (36.7%)	356 (21.5%)	315 (19.1%)	377 (22.8%)
2 g/day reduction	1208 (36.5%)	710 (21.5%)	628 (19.0%)	755 (22.8%)
**Lifetime**				
SDT 5 g/day achieved	67,808 (33.9%)	38,074 (19.1%)	41,044 (20.6%)	51,923 (26.1%)
NPHS 30% reduction	59,850 (35.1%)	31,795 (18.6%)	33,629 (19.8%)	45,149 (26.5%)
20% reduction	39,998 (35.2%)	21,212 (18.6%)	22,510 (19.8%)	29,964 (26.3%)
15% reduction	30,063 (35.2%)	15,926 (18.7%)	16,896 (19.8%)	22,450 (26.3%)
1 g/day reduction	26,477 (35.7%)	13,624 (18.4%)	14,302 (19.3%)	19,645 (26.6%)
2 g/day reduction	52,584 (35.6%)	27,222 (18.4%)	28,530 (19.3%)	39,433 (26.7%)

Note: Numbers in the table are absolute number of HALYs gained by CKD cause over different time horizons, with percentage contribution to total HALYs gained per scenario. SDT, Australian Suggested Dietary Target; NPHS, National Preventive Health Strategy, 2021–2030 target; CKD HTN, chronic kidney disease due to hypertension; CKD DM, chronic kidney disease due to diabetes mellitus; CKD GMN, chronic kidney disease due to glomerulonephritis; CKD Other, chronic kidney disease due to other or unspecified causes.

## Data Availability

The datasets supporting the analysis and conclusions of this article are included within the article and supporting Supplementary material files. These data were obtained from publicly available repositories and sites including the Global Burden of Disease Results tool (https://ghdx.healthdata.org/gbd-results-tool (accessed on 10 January 2022)), the Australian Bureau of Statistics National Health Survey 2017–2018 (https://www.abs.gov.au/statistics/health/health-conditions-and-risks/national-health-survey-first-results/latest-release (accessed on 10 January 2022)) and the National Nutrition and Physical Activity Survey 2011–2012 (https://www.abs.gov.au/statistics/microdata-tablebuilder/available-microdata-tablebuilder/australian-health-survey-nutrition-and-physical-activity (accessed on 10 January 2022)).

## Supplementary Materials

The following supporting information can be downloaded at: https://www.mdpi.com/article/10.3390/nu15020318/s1, Figure S1: Conceptual Markov model for chronic kidney disease; Figure S2: Framework of the proportional multistate life table model [52], including the interaction with the CKD Markov models and the risk factor (blood pressure); Figure S3: Reduction in cumulative number of new CKD cases (panel a, men; panel b, women) and deaths (panel c, men; panel d, women) by CKD cause, between 2019 to 2030; Figure S4: Reduction in cumulative number of new CKD cases (panel a, men; panel b, women) and deaths (panel c, men; panel d, women) by CKD cause over the remaining lifetime of the 2019 Australian population; Figure S5: Sex-specific relative reduction in incidence of CKD by cause between 2019 to 2030 for all six scenarios; Figure S6: Lifetime sex-specific relative reductions in incidence of CKD by cause for all six scenarios; Figure S7: Sex-specific relative reduction in mortality from CKD by cause for all six scenarios between 2019 to 2030; Figure S8: Lifetime sex-specific relative reduction in mortality from CKD by cause for all six scenarios; Figure S9: Overall and sex-specific HALYs (discounted at 3%) gained between 2019 to 2030 (panel A) and over the lifetime (panel B) of the 2019 adult population; Figure S10: Healthcare costs savings (discounted at 5%) between 2019 to 2030 by CKD cause and gender; Figure S11: Healthcare costs savings (discounted at 5%) by CKD cause and gender over the lifetime of the 2019 adult population; Table S1: Mean daily salt intake among Australian adults; Table S2: Age- and sex-specific systolic blood pressure distribution of adult Australians; Table S3: Relative risks of CKD per 10 mmHg increase in systolic blood pressure; Table S4: Age-specific incidence, prevalence and case fatality rates in 2019 for men by CKD cause in Australia; Table S5: Age-specific incidence, prevalence and case fatality rates in 2019 for women by CKD cause in Australia; Table S6: Age- and sex-specific disability weights for each CKD cause and overall background disability weights for adult Australians in 2019; Table S7: Age- and sex-specific population numbers, all-cause mortality rates and costs per CKD for adult Australians in 2019 [52,53,54].
