# Supplementary material for: Estimated Impact of Achieving the Australian National Sodium Reduction Targets on Blood Pressure, Chronic Kidney Disease Burden and Healthcare Costs: A Modelling Study
Source: Nutrients. 2023 Jan 9;15(2):318. doi: 10.3390/nu15020318 (PMC9865653; doi:10.3390/nu15020318)
Supplement: Supplementary file 1 [file nutrients-15-00318-s001.zip › nutrients-2125422-supplementary.pdf]

Supplemental Materials

Estimated Impact of Achieving the Australian National Sodium Reduction Targets on Blood Pressure, Chronic Kidney Disease Burden and Healthcare Costs: A Modelling Study

Leopold Ndemnge Aminde *, Mary Njeri Wanjau, Linda J. Cobiac and J. Lennert Veerman

Public Health & Economics Modelling Group, School of Medicine & Dentistry, Griffith University, Gold Coast, QLD 4222, Australia

* Correspondence: l.aminde@griffith.edu.au

Contents

1. Additional methods description 2
   1.1. Estimating risk of CKD based on shifts in systolic blood pressure distribution 2
   1.2. Chronic kidney disease epidemiology and Markov models 3
   1.3. The proportional multi-cohort multistate life table model 4
2. Input data 6
   2.1. Salt intake 6
   2.2. Systolic blood pressure distribution 7
   2.3. Relative risks of CKD from elevated blood pressure 8
   2.4. Baseline CKD epidemiological data 9
   2.5. Disability weights by CKD cause and overall population disability weights 11
   2.6. Population numbers, all-cause mortality rates and healthcare costs 12
3. Additional results 13

1. Additional methods description

1.1. Estimating risk of CKD based on shifts in systolic blood pressure distribution

To model the effect of the sodium reduction interventions on systolic blood pressure (SBP) and subsequent CKD, we used the potential impact fraction (PIF) – an epidemiological measure that quantifies the proportional change in disease incidence (or mortality) that results from a change in exposure to a risk factor – to estimate changes in CKD incidence. We modelled blood pressure as a continuous variable (assuming a normal distribution) using the 'distribution shift' method [29]. This PIF calculation includes integrating SBP distributions with the relative risk function for each 5-year age-group and sex. The theoretical minimum risk exposure level for SBP is assumed to be 115 mmHg, which is the optimal level of blood pressure which is the lowest level considered for elevated vascular risk. This is based on evidence from robust individual participant data meta-analysis of large prospective cohort studies [30].

The effects of sodium reduction on blood pressure are known to vary with baseline blood pressure, with steeper gradient in people with higher baseline blood pressures. Given this difference, we modelled a differential impact of changes in sodium on systolic BP in people with and without hypertension and modified in the PIF formula below. These PIF calculations were done separately for each of the CKD causes (CKD due to hypertension, CKD due diabetes mellitus, CKD due to glomerulonephritis, CKD due to other or unspecified causes).

$$PIF = \frac{\int_a^b RR(x)P(x)dx - \left(\int_{a1}^{b1} RR(x)P*(x)dx + \int_{a2}^{b2} RR(x)P*(x)dx\right)}{\int_a^b RR(x)P(x)dx}$$

Where:

x = SBP exposure levels, RR(x) = relative risk function, P(x) = original SBP distribution, P* = SBP distribution without the intervention, a = start integration limit (90mmHg), b = end integration limit (220mmHg), a1 = start integration limit for normotensive people (90mmHg), b1 = end integration limit for normotensive people (139.9mmHg), a2 = start integration limit for hypertensive people (140mmHg), b2 = end integration limit for hypertensive people (220mmHg).

Post intervention incidence (I*) of CKD is, I* = I x (1-PIF).

Where: I = current incidence of CKD in the Australian population by sex and five-year age groups.

I* = new incidence of CKD after the intervention.

1.2. Chronic kidney disease epidemiology and Markov models

We developed separate Markov sub-models for each of the four main CKD causes included in our analysis, that is, CKD due to hypertension, CKD due to diabetes mellitus, CKD due to glomerulonephritis and CKD due to other or unspecified causes, as implemented in the GBD study [31]. For baseline epidemiological data on each of the CKD causes, we used Australia specific estimates of incidence, prevalence and mortality from the Global Burden of Disease 2019 study [11]. Each disease (CKD cause) Markov model has four distinct health states: healthy (alive without CKD), diseased (alive with CKD), death from (any of the) CKDs and death due to other causes, with death being an absorbing state.

The movement of proportions of the populations between these health states is governed by transition hazards or probabilities (incidence, case fatality, remission), which are estimated from incidence and case fatality rates. The latter are seldom available in epidemiological studies. As such, we used the DISMOD-II epidemiological software [32] to derive CKD case fatality rates for each of the CKD causes by sex and five-year age-group cohort. With a minimum of three disease epidemiological input parameters, and data on overall population numbers and background mortality rates by disaggregated by sex and age-groups, this analytical software uses a set of differential equations that exploit the causal relation in a typical disease process to estimate absent epidemiological parameters (such as case fatality in this instance) while maintaining stability in the overall disease epidemiology [32].

Given the chronic and progressive nature of CKD, remission was assumed to be zero in our Markov models. For all four CKD causes modelled, the starting population in the diseased state of the Markov models consisted of the proportion of prevalent CKD cases in the population for each sex and five-year age-group, and the rest were allocated to the ‘healthy’ state. For these models, a 1-year cycle length was used for state membership and the Markov models were linked to the life table. The figure below depicts the CKD state-transition disease models.

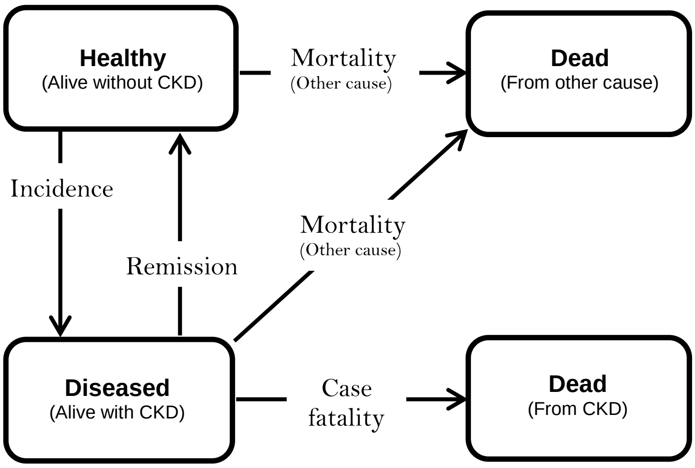

Figure S1: Conceptual Markov model for chronic kidney disease (adapted from [32])

1.3. The proportional multi-cohort multistate life table model

A proportional multi-cohort multistate life table model was developed to estimate the difference in life years lived by the Australian population aged 25 years and above. We compared a population that continues to consume sodium at current levels with an identical 'intervention' population that achieves the sodium reduction targets. The multistate lifetable has the ability to deal with comorbidity with each of the diseases is assumed to be independent [52]. The population is stratified in multiple cohorts by sex and five-year age groups. Disease-specific morbidity and mortality rates from the Markov models with and without the sodium reduction influence the all-cause disability and background mortality rates (hence, dealing with competing mortality risks) in the lifetable. The calculated years lived by each of the cohorts is adjusted for sub-optimal health or disability (a numerical quantification of the degree of health loss due to a disease) using estimates derived from the GBD 2019 study [11].

For each of the CKDs modelled, this sub-optimal health adjustment is estimated by dividing the sex and age-specific years lived with disability (YLD) for that disease by the corresponding prevalence numbers [11]. This gives an average weight of the proportion of health knocked out by the disease. This 'disability' weight is further adjusted for poor health due to background

diseases not included in the model using all-cause prevalent years lived with disability (pYLD) per capita (after excluding the diseases modelled). This generates health-adjusted life years (HALY), where one HALY is equivalent to a year of life in perfect health [53]. The difference in HALYs between the populations receiving the sodium reduction intervention and those not receiving is indicative of the health benefit attributed to the intervention. All the population cohorts are simulated for their remaining lifetime till they reach 100 years or die.

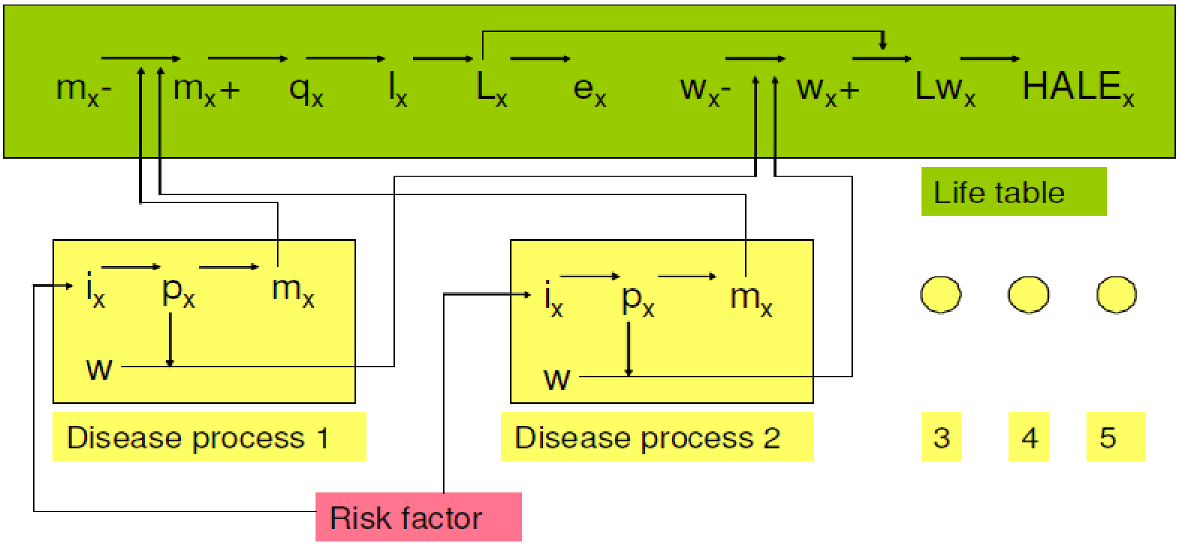

Figure S2: Framework of the proportional multistate life table model [52], including the interaction with the CKD Markov models and the risk factor (blood pressure).
In the disease Markov models (yellow); i = incidence, p = prevalence, m = mortality.
In the life table (green); m = mortality, q = mortality probability, I = number of survivors, L = life years, Lw = health-adjusted life years (HALY), HALE = health-adjusted life expectancy, x = age. '-': parameter excluding the modelled diseases, '+': overall parameter (including the diseases modelled).

2. Input data

2.1. Salt intake

Dietary salt intake estimates were obtained from an Australian meta-analysis [23] that included 31 published and 1 unpublished dataset of studies conducted across Australia between 1989 and 2015. This meta-analysis included the National Nutrition and Physical Activity Survey (NNPAS) 2011-12 [24] from which we derived the age pattern of sodium consumption and used to adjust the overall gender-specific estimates accordingly. Estimates are weighted mean salt intake obtained from 24-hour urine collections after adjusting for non-urinary salt excretion.

Table S1: Mean daily salt intake among Australian adults

	Men		Women	
**Age groups**	**Mean**	**95% CI**	**Mean**	**95% CI**
**19-30 years**	11.68	11.23 – 12.13	8.24	7.84 – 8.65
**31-50 years**	10.91	10.49 – 11.33	7.71	7.33 – 8.09
**51-70 years**	9.39	9.03 – 9.76	7.06	6.71 – 7.41
**71+ years**	8.30	7.98 – 8.62	6.35	6.04 – 6.66
**Overall**	10.07	9.68 – 10.46	7.34	6.98 – 7.70

CI, confidence interval.

2.2. Systolic blood pressure distribution

Systolic blood pressure data were obtained from the Australian National Health Survey, 2017-18 [25, 26]. This nationally representative survey used a random multistage area sampling of private dwellings, with a final sample of 16,384 dwellings and 21,315 participants. Consenting adults (18 years and above) typically had two BP measurements taken, and the second reading was used. When a third reading was obtained, the average of the second and third readings was recorded as the measured BP, unless the third reading differed by more than 20 mmHg. Measurement was considered invalid if all three BP readings differed by 20 mmHg or more [25, 26]. This survey reported mean blood pressures, the proportion of people with hypertension and the proportion of people in various blood pressure ranges, e.g., <100mmHg, 100 to ≤110 mmHg, etc. together with the relative standard error (RSE) of the estimates. We used the RSE to back-calculate the standard errors, and assuming a normal distribution, we used the proportions of people in various blood pressure categories to derive the variance and standard deviations (SD).

Table S2: Age- and sex-specific systolic blood pressure distribution of adult Australians

	**Men**		**Women**	
**Age group**	**Mean (SD)**	**RSE, %**	**Mean (SD)**	**RSE, %**
**18 – 24 years**	119.5 (18.2)	0.7	107.6 (14.7)	0.5
**25 – 34 years**	120.3 (13.5)	0.4	108.5 (15.7)	0.4
**35 – 44 years**	121.3 (11.6)	0.4	112.5 (15.7)	0.4
**45 – 54 years**	126.5 (17.9)	0.4	119.6 (17.0)	0.5
**55 – 64 years**	132.4 (14.7)	0.5	126.8 (20.8)	0.5
**65 – 74 years**	134.9 (21.3)	0.5	133.6 (17.7)	0.5
**75 – 84 years**	136.5 (20.9)	0.7	137.9 (17.0)	0.6
**85+ years**	140.2 (33.3)	1.3	140.8 (28.0)	1.2
**Overall**	126.1 (16.8)	0.2	119.2 (21.0)	0.2

SD, standard deviation; RSE, relative standard error.

2.3. Relative risks of CKD from elevated blood pressure

Estimates of the relative risks (and 95% uncertainty intervals) of developing chronic kidney disease (CKD) from high blood pressure were obtained from the Global Burden of Disease 2019 study [31]. These are reported to represent the risk of morbidity/mortality of either form of CKD for both men and women for every 10mmHg increase in systolic blood pressure. We used these relative risks to model the risk of incidence of the different forms of CKD due to increased systolic blood pressure. CKD-HTN, chronic kidney disease due to hypertension; CKD-DM, chronic kidney disease due to diabetes mellitus; CKD-GMN, chronic kidney disease due to glomerulonephritis; CKD-U, chronic kidney disease due to other or unspecified causes.

Table S3: Relative risks of CKD per 10mmHg increase in systolic blood pressure

Age	CKD-HTN	CKD-DM	CKD-GMN	CKD-U
**25-29yrs**	1.281 (1.180 – 1.385)	1.283 (1.186 – 1.397)	1.281 (1.182 to 1.383)	1.282 (1.181 to 1.395)
**30-34yrs**	1.281 (1.180 – 1.385)	1.283 (1.186 – 1.397)	1.281 (1.182 to 1.383)	1.282 (1.181 to 1.395)
**35-39yrs**	1.281 (1.180 – 1.385)	1.283 (1.186 – 1.397)	1.281 (1.182 to 1.383)	1.282 (1.181 to 1.395)
**40-44yrs**	1.281 (1.180 – 1.385)	1.283 (1.186 – 1.397)	1.281 (1.182 to 1.383)	1.282 (1.181 to 1.395)
**45-49yrs**	1.281 (1.180 – 1.385)	1.283 (1.186 – 1.397)	1.281 (1.182 to 1.383)	1.282 (1.181 to 1.395)
**50-54yrs**	1.281 (1.180 – 1.385)	1.283 (1.186 – 1.397)	1.281 (1.182 to 1.383)	1.282 (1.181 to 1.395)
**55-59yrs**	1.281 (1.180 – 1.385)	1.283 (1.186 – 1.397)	1.281 (1.182 to 1.383)	1.282 (1.181 to 1.395)
**60-64yrs**	1.281 (1.180 – 1.385)	1.283 (1.186 – 1.397)	1.281 (1.182 to 1.383)	1.282 (1.181 to 1.395)
**65-69yrs**	1.281 (1.180 – 1.385)	1.283 (1.186 – 1.397)	1.281 (1.182 to 1.383)	1.282 (1.181 to 1.395)
**70-74yrs**	1.281 (1.180 – 1.385)	1.283 (1.186 – 1.397)	1.281 (1.182 to 1.383)	1.282 (1.181 to 1.395)
**75-79yrs**	1.281 (1.180 – 1.385)	1.283 (1.186 – 1.397)	1.281 (1.182 to 1.383)	1.282 (1.181 to 1.395)
**80-84yrs**	1.281 (1.180 – 1.385)	1.283 (1.186 – 1.397)	1.281 (1.182 to 1.383)	1.282 (1.181 to 1.395)
**85-89yrs**	1.281 (1.180 – 1.385)	1.283 (1.186 – 1.397)	1.281 (1.182 to 1.383)	1.282 (1.181 to 1.395)
**90-94yrs**	1.281 (1.180 – 1.385)	1.283 (1.186 – 1.397)	1.281 (1.182 to 1.383)	1.282 (1.181 to 1.395)
**95+yrs**	1.281 (1.180 – 1.385)	1.283 (1.186 – 1.397)	1.281 (1.182 to 1.383)	1.282 (1.181 to 1.395)

2.4. Baseline CKD epidemiological data

Australian estimates of CKD incidence, prevalence and mortality by cause were obtained from the Global Burden of Disease 2019 study [11, 31]. We report outputs after modelling through the DISMOD-II epidemiological software [32] to obtain case fatality rates. DISMOD-II applies causal differential equations to estimate case fatality rates that were unavailable, while preserving consistency in the overall disease epidemiology. Numbers in the table below are rates per 1.

Table S4: Age-specific incidence, prevalence and case fatality rates in 2019 for men by CKD cause in Australia

Cause	CKD-HTN			CKD-DM			CKD-GMN			CKD-U		
**Age groups**	**Incidence**	**Prevalence**	**Case fatality**	**Incidence**	**Prevalence**	**Case fatality**	**Incidence**	**Prevalence**	**Case fatality**	**Incidence**	**Prevalence**	**Case fatality**
**25-29yrs**	0.0000	0.0004	0.0001	0.0002	0.0014	0.0000	0.0000	0.0018	0.0006	0.0013	0.0189	0.0000
**30-34yrs**	0.0000	0.0005	0.0002	0.0005	0.0034	0.0000	0.0000	0.0018	0.0009	0.0013	0.0252	0.0000
**35-39yrs**	0.0001	0.0008	0.0002	0.0004	0.0059	0.0000	0.0001	0.0020	0.0016	0.0013	0.0317	0.0001
**40-44yrs**	0.0001	0.0011	0.0004	0.0004	0.0079	0.0000	0.0001	0.0022	0.0026	0.0010	0.0374	0.0001
**45-49yrs**	0.0001	0.0016	0.0006	0.0005	0.0102	0.0001	0.0001	0.0024	0.0039	0.0010	0.0416	0.0002
**50-54yrs**	0.0002	0.0024	0.0008	0.0007	0.0132	0.0002	0.0001	0.0026	0.0057	0.0018	0.0481	0.0002
**55-59yrs**	0.0003	0.0036	0.0012	0.0011	0.0175	0.0003	0.0001	0.0029	0.0088	0.0026	0.0581	0.0003
**60-64yrs**	0.0006	0.0058	0.0018	0.0017	0.0241	0.0004	0.0001	0.0031	0.0141	0.0048	0.0742	0.0004
**65-69yrs**	0.0016	0.0108	0.0023	0.0030	0.0350	0.0006	0.0002	0.0034	0.0218	0.0114	0.1083	0.0006
**70-74yrs**	0.0034	0.0225	0.0030	0.0052	0.0540	0.0008	0.0003	0.0040	0.0346	0.0239	0.1807	0.0007
**75-79yrs**	0.0059	0.0444	0.0042	0.0055	0.0800	0.0011	0.0003	0.0045	0.0609	0.0398	0.3010	0.0009
**80-84yrs**	0.0076	0.0752	0.0067	0.0030	0.0993	0.0017	0.0005	0.0044	0.1229	0.0489	0.4423	0.0013
**85-89yrs**	0.0074	0.1060	0.0123	0.0005	0.1056	0.0032	0.0010	0.0037	0.2952	0.0410	0.5563	0.0022
**90-94yrs**	0.0061	0.1264	0.0233	0.0000	0.1039	0.0066	0.0016	0.0025	0.7195	0.0167	0.6130	0.0037
**95+yrs**	0.0068	0.1363	0.0361	0.0000	0.0997	0.0125	0.0026	0.0020	1.3292	0.0063	0.6262	0.0057

CKD-HTN, Chronic kidney disease due to hypertension; CKD-DM, Chronic kidney disease due to diabetes mellitus; CKD-GMN, Chronic kidney disease due to glomerulonephritis; CKD-U, Chronic kidney disease due to other or unspecified causes.

Table S5: Age-specific incidence, prevalence and case fatality rates in 2019 for women by CKD cause in Australia

Cause	CKD-HTN			CKD-DM			CKD-GMN			CKD-U		
Age groups	Incidence	Prevalence	Case fatality	Incidence	Prevalence	Case fatality	Incidence	Prevalence	Case fatality	Incidence	Prevalence	Case fatality
**25-29yrs**	0.0000	0.0004	0.0001	0.0003	0.0019	0.0000	0.0000	0.0013	0.0006	0.0023	0.0271	0.0000
**30-34yrs**	0.0000	0.0006	0.0001	0.0006	0.0042	0.0000	0.0000	0.0015	0.0009	0.0020	0.0376	0.0000
**35-39yrs**	0.0001	0.0008	0.0001	0.0005	0.0070	0.0000	0.0000	0.0016	0.0014	0.0015	0.0455	0.0001
**40-44yrs**	0.0001	0.0011	0.0002	0.0004	0.0092	0.0000	0.0000	0.0018	0.0019	0.0012	0.0515	0.0001
**45-49yrs**	0.0001	0.0016	0.0003	0.0004	0.0113	0.0001	0.0000	0.0019	0.0028	0.0012	0.0563	0.0001
**50-54yrs**	0.0002	0.0024	0.0004	0.0007	0.0139	0.0001	0.0000	0.0019	0.0044	0.0022	0.0641	0.0002
**55-59yrs**	0.0004	0.0037	0.0007	0.0011	0.0181	0.0002	0.0000	0.0020	0.0072	0.0038	0.0774	0.0002
**60-64yrs**	0.0007	0.0062	0.0010	0.0018	0.0250	0.0003	0.0000	0.0021	0.0114	0.0075	0.1015	0.0004
**65-69yrs**	0.0017	0.0118	0.0013	0.0029	0.0360	0.0004	0.0001	0.0022	0.0169	0.0167	0.1518	0.0004
**70-74yrs**	0.0032	0.0234	0.0016	0.0045	0.0533	0.0005	0.0001	0.0025	0.0274	0.0325	0.2478	0.0005
**75-79yrs**	0.0047	0.0422	0.0024	0.0044	0.0749	0.0008	0.0002	0.0026	0.0526	0.0515	0.3897	0.0007
**80-84yrs**	0.0054	0.0657	0.0044	0.0022	0.0897	0.0013	0.0002	0.0025	0.1187	0.0619	0.5431	0.0010
**85-89yrs**	0.0049	0.0876	0.0095	0.0004	0.0942	0.0027	0.0005	0.0019	0.3238	0.0492	0.6565	0.0017
**90-94yrs**	0.0041	0.1011	0.0213	0.0000	0.0927	0.0065	0.0010	0.0012	0.8617	0.0168	0.7066	0.0033
**95+yrs**	0.0052	0.1074	0.0365	0.0000	0.0887	0.0136	0.0015	0.0009	1.6273	0.0060	0.7151	0.0053

CKD-HTN, Chronic kidney disease due to hypertension; CKD-DM, Chronic kidney disease due to diabetes mellitus; CKD-GMN, Chronic kidney disease due to glomerulonephritis; CKD-U, Chronic kidney disease due to other or unspecified causes.

2.5. Disability weights by CKD cause and overall population disability weights

To adjust life years gained for poor quality life due to CKD, we obtained Australian specific estimates of years lived with disability (YLD) and prevalence numbers for each CKD cause from the Global Burden of Disease 2019 study [11, 31]. We divided the sex and age-specific YLD by the corresponding prevalent CKD cases. This gives an average disability weight which reflects the proportion of the quality of life knocked out by the disease. This disability weight is further adjusted for poor health due to background diseases not included in the model using all-cause prevalent years lived with disability (pYLD) per capita (after excluding the diseases modelled). The table below depicts the age- and sex-specific disability weights used in the model.

Table S6: Age- and sex-specific disability weights for each CKD cause and overall background disability weights for adult Australians in 2019

**Age**	**CKD-HTN**		**CKD-DM**		**CKD-GMN**		**CKD-U**		**All-cause pYLD rate**	
	**Men**	**Women**	**Men**	**Women**	**Men**	**Women**	**Men**	**Women**	**Men**	**Women**
**25-29yrs**	0.1267	0.1355	0.0015	0.0018	0.0644	0.0862	0.0063	0.0070	0.1081	0.1338
**30-34yrs**	0.1176	0.1252	0.0022	0.0026	0.0765	0.0961	0.0058	0.0065	0.1142	0.1391
**35-39yrs**	0.0998	0.1142	0.0032	0.0041	0.0873	0.0999	0.0054	0.0066	0.1207	0.1455
**40-44yrs**	0.0923	0.1009	0.0054	0.0063	0.0971	0.1143	0.0056	0.0058	0.1269	0.1532
**45-49yrs**	0.0799	0.0794	0.0091	0.0099	0.1096	0.1107	0.0066	0.0063	0.1329	0.1580
**50-54yrs**	0.0730	0.0618	0.0125	0.0124	0.1186	0.1199	0.0071	0.0068	0.1434	0.1642
**55-59yrs**	0.0661	0.0505	0.0157	0.0150	0.1273	0.1231	0.0073	0.0068	0.1603	0.1757
**60-64yrs**	0.0536	0.0375	0.0173	0.0159	0.1309	0.1209	0.0072	0.0064	0.1823	0.1921
**65-69yrs**	0.0415	0.0284	0.0172	0.0163	0.1372	0.1217	0.0069	0.0058	0.2115	0.2148
**70-74yrs**	0.0337	0.0253	0.0167	0.0161	0.1249	0.1230	0.0063	0.0056	0.2438	0.2422
**75-79yrs**	0.0298	0.0232	0.0167	0.0163	0.1110	0.1214	0.0065	0.0062	0.2745	0.2730
**80-84yrs**	0.0299	0.0238	0.0186	0.0170	0.1038	0.1209	0.0082	0.0075	0.3069	0.3123
**85-89yrs**	0.0290	0.0240	0.0185	0.0170	0.0904	0.1218	0.0104	0.0092	0.3385	0.3538
**90-94yrs**	0.0270	0.0221	0.0168	0.0154	0.0940	0.1105	0.0116	0.0099	0.3692	0.4013
**95+yrs**	0.0309	0.0243	0.0176	0.0243	0.0965	0.1102	0.0158	0.0129	0.4117	0.4574

CKD-HTN, Chronic kidney disease due to hypertension; CKD-DM, Chronic kidney disease due to diabetes mellitus; CKD-GMN, Chronic kidney disease due to glomerulonephritis; CKD-U, Chronic kidney disease due to other or unspecified causes; pYLD, overall prevalent years lived with disability

2.6. Population numbers, all-cause mortality rates and healthcare costs

Population and mortality data are from the Australian Bureau of Statistics [54]. Costs estimates were calculated using data from the Australian Institute of Health and Welfare (AIHW) health expenditure for Australia, 2018-2019 [33]. The AIHW pools data from multiple hospital, morbidity, non-admitted patient, and pharmaceutical datasets covering public, private and primary care services including general practitioner and allied health services, pathology and medical imaging, patient admissions, emergency and outpatient services, out-of-hospital services and prescription pharmaceuticals from across the country. It maps these to specific areas of expenditure and to disease conditions using ICD-10-AM codes while implementing a predominantly top-down costing approach. Further details are reported elsewhere (34).

Table S7: Age- and sex-specific population numbers, all-cause mortality rates and costs per CKD for adult Australians in 2019.

	**Men**			**Women**		
**Age group**	**Population numbers**	**Mortality rate**	**Cost per prevalent CKD**	**Population numbers**	**Mortality rate**	**Cost per prevalent CKD**
**25-29yrs**	957,746	0.0007	AU$ 249	949,146	0.0003	AU$ 168
**30-34yrs**	933,587	0.0009	AU$ 246	959,375	0.0004	AU$ 166
**35-39yrs**	885,449	0.0011	AU$ 239	896,946	0.0006	AU$ 200
**40-44yrs**	793,623	0.0016	AU$ 294	802,591	0.0009	AU$ 220
**45-49yrs**	825,686	0.0022	AU$ 387	854,071	0.0013	AU$ 267
**50-54yrs**	750,782	0.0033	AU$ 429	785,017	0.0020	AU$ 280
**55-59yrs**	757,941	0.0050	AU$ 469	790,197	0.0030	AU$ 251
**60-64yrs**	677,332	0.0076	AU$ 468	714,617	0.0046	AU$ 232
**65-69yrs**	596,458	0.0121	AU$ 498	631,045	0.0072	AU$ 243
**70-74yrs**	518,927	0.0201	AU$ 371	539,265	0.0122	AU$ 181
**75-79yrs**	351,089	0.0344	AU$ 358	383,275	0.0220	AU$ 172
**80-84yrs**	227,926	0.0617	AU$ 323	277,175	0.0425	AU$ 156
**85-89yrs**	129,208	0.1103	AU$ 309	184,648	0.0830	AU$ 150
**90-94yrs**	53,978	0.1172	AU$ 309	99,014	0.1524	AU$ 150
**95+ yrs**	13,876	0.2117	AU$ 309	34,246	0.2209	AU$ 150

CKD, chronic kidney disease; AU$, 2019 Australian dollars.

3. Additional results

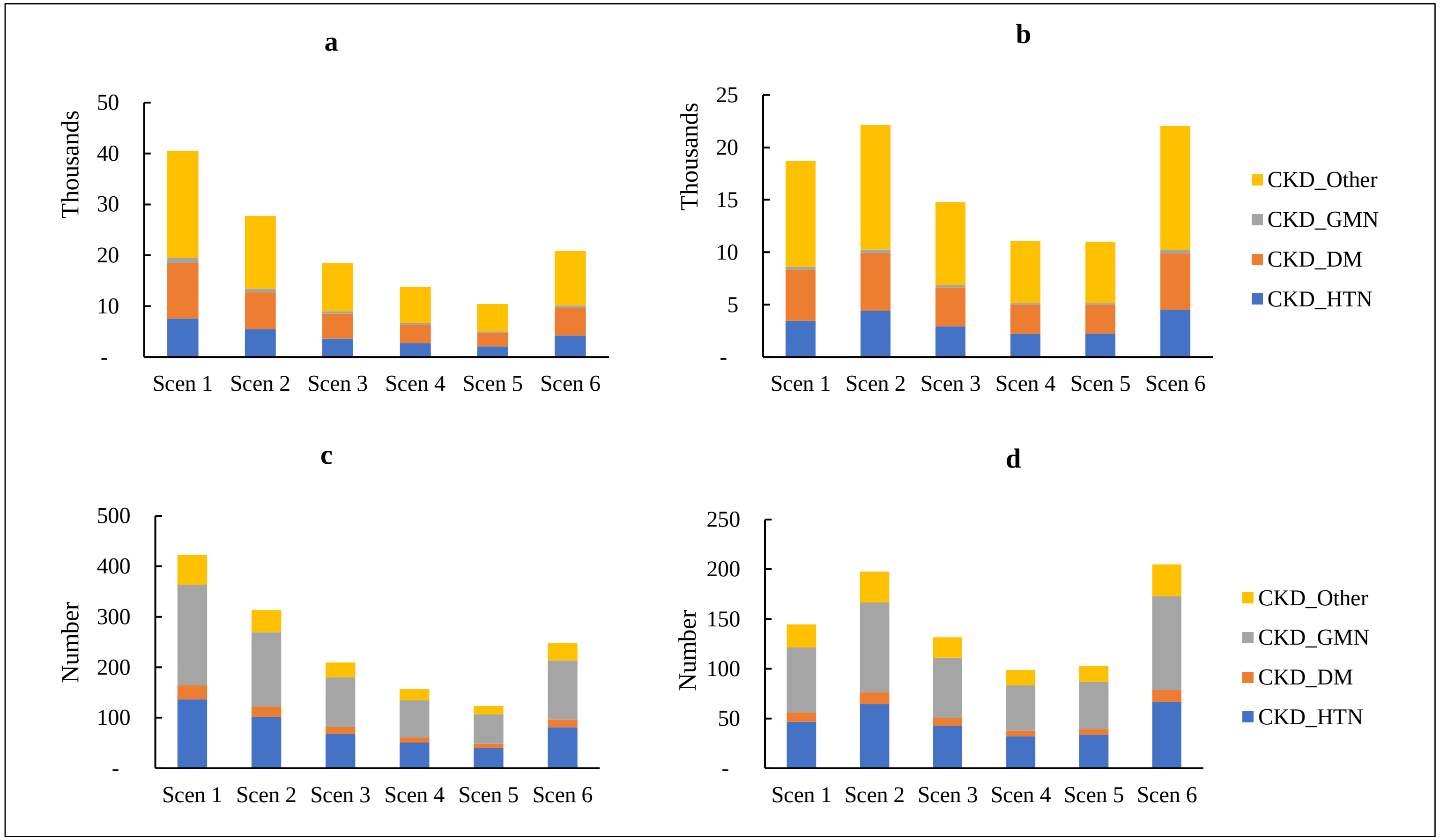

Figure S3: Reduction in cumulative number of new CKD cases (panel a, men; panel b, women) and deaths (panel c, men; panel d, women) by CKD cause, between 2019 to 2030.

Scenario 1: Reducing current salt intake to an average of 5 g/day (Australian Suggested Dietary Target), Scenario 2: 30% relative reduction in current salt intake (National Preventive Health Strategy, 2021-2030 target), Scenario 3: 20% relative reduction in current salt intake, Scenario 4: 15% relative reduction in current salt intake; Scenario 5: reduction in current salt intake by 1g; Scenario 6: reduction in current salt intake by 2g.

Scen, Scenario; CKD_HTN, chronic kidney disease due to hypertension; CKD_DM, chronic kidney disease due to diabetes mellitus; CKD_GMN, chronic kidney disease due to glomerulonephritis; CKD_Other, chronic kidney disease due to other or unspecified causes.

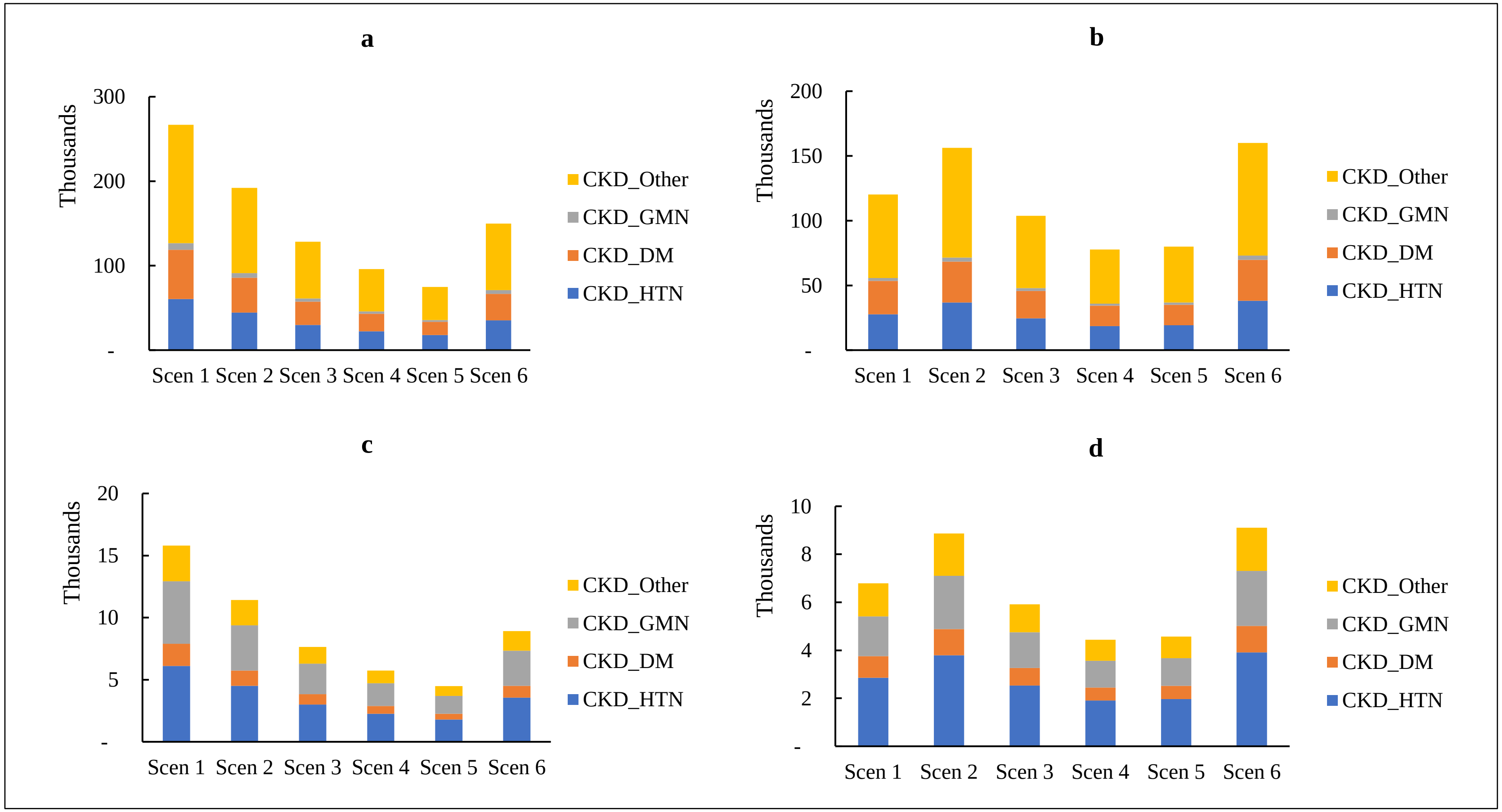

Figure S4: Reduction in cumulative number of new CKD cases (panel a, men; panel b, women) and deaths (panel c, men; panel d, women) by CKD cause over the remaining lifetime of the 2019 Australian population.

Scenario 1: Reducing current salt intake to an average of 5 g/day (Australian Suggested Dietary Target), Scenario 2: 30% relative reduction in current salt intake (National Preventive Health Strategy, 2021-2030 target), Scenario 3: 20% relative reduction in current salt intake, Scenario 4: 15% relative reduction in current salt intake; Scenario 5: reduction in current salt intake by 1g; Scenario 6: reduction in current salt intake by 2g.

Scen, Scenario; CKD_HTN, chronic kidney disease due to hypertension; CKD_DM, chronic kidney disease due to diabetes mellitus; CKD_GMN, chronic kidney disease due to glomerulonephritis; CKD_Other, chronic kidney disease due to other or unspecified causes.

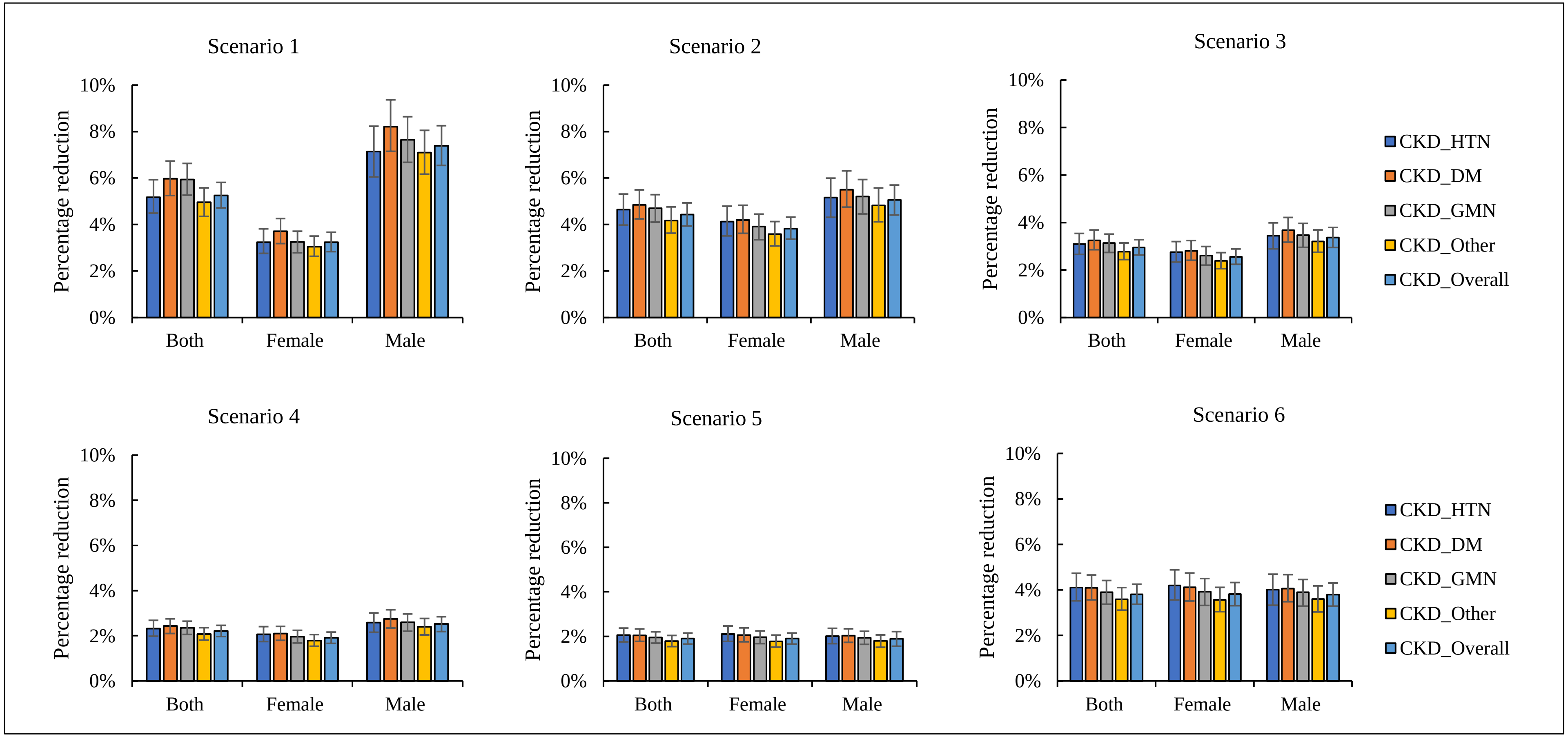

Figure S5: Sex-specific relative reduction in incidence of CKD by cause between 2019 to 2030 for all six scenarios.
Scenario 1: Reducing current salt intake to an average of 5 g/day (Australian Suggested Dietary Target), Scenario 2: 30% relative reduction in current salt intake (National Preventive Health Strategy, 2021-2030 target), Scenario 3: 20% relative reduction in current salt intake, Scenario 4: 15% relative reduction in current salt intake; Scenario 5: reduction in current salt intake by 1g; Scenario 6: reduction in current salt intake by 2g. Error bars reflect the 95% uncertainty intervals.
Scen, Scenario; CKD_HTN, chronic kidney disease due to hypertension; CKD_DM, chronic kidney disease due to diabetes mellitus; CKD_GMN, chronic kidney disease due to glomerulonephritis; CKD_Other, chronic kidney disease due to other or unspecified causes.

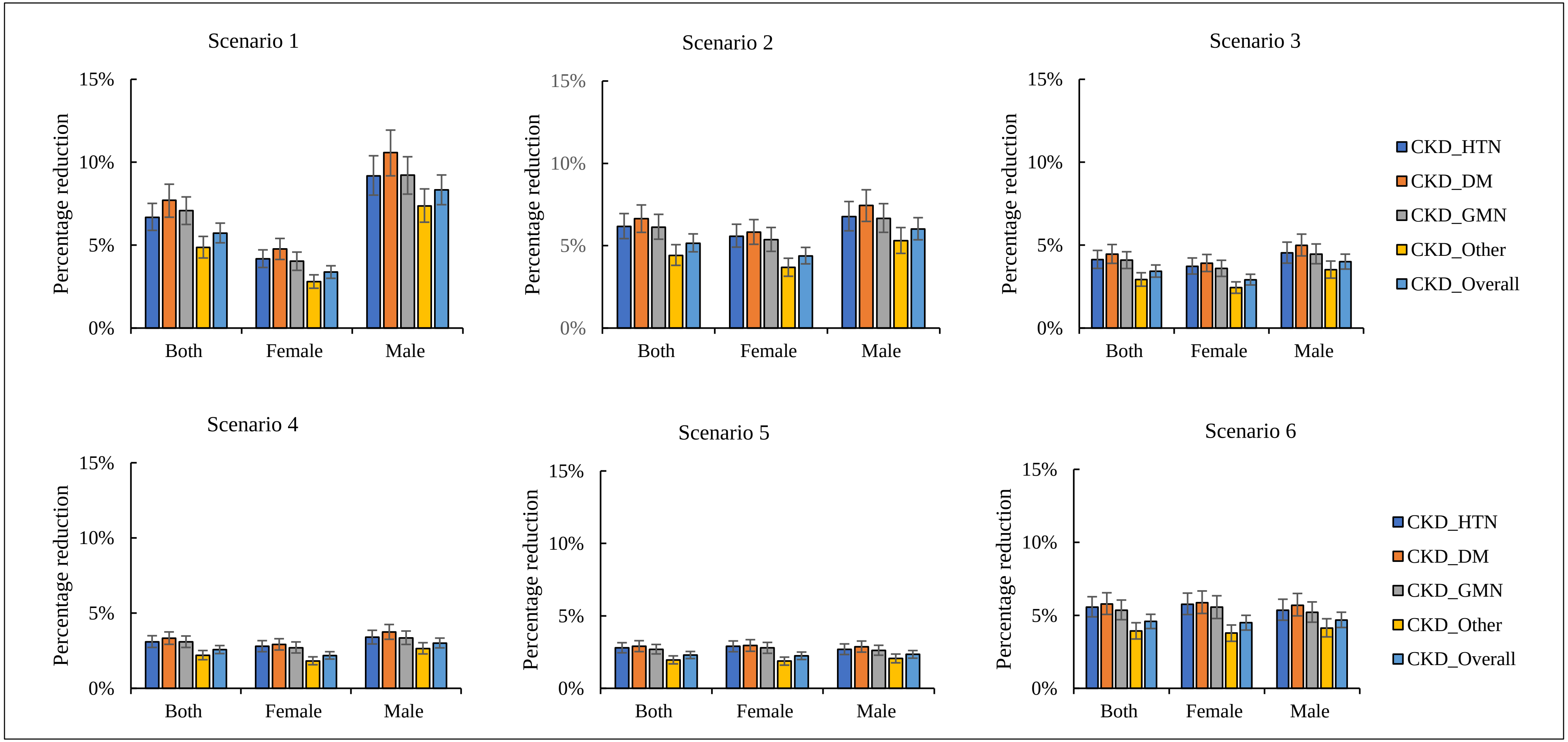

Figure S6: Lifetime sex-specific relative reductions in incidence of CKD by cause for all six scenarios

Scenario 1: Reducing current salt intake to an average of 5 g/day (Australian Suggested Dietary Target), Scenario 2: 30% relative reduction in current salt intake (National Preventive Health Strategy, 2021-2030 target), Scenario 3: 20% relative reduction in current salt intake, Scenario 4: 15% relative reduction in current salt intake; Scenario 5: reduction in current salt intake by 1g; Scenario 6: reduction in current salt intake by 2g. Error bars reflect the 95% uncertainty intervals.

Scen, Scenario; CKD_HTN, chronic kidney disease due to hypertension; CKD_DM, chronic kidney disease due to diabetes mellitus; CKD_GMN, chronic kidney disease due to glomerulonephritis; CKD_Other, chronic kidney disease due to other or unspecified causes.

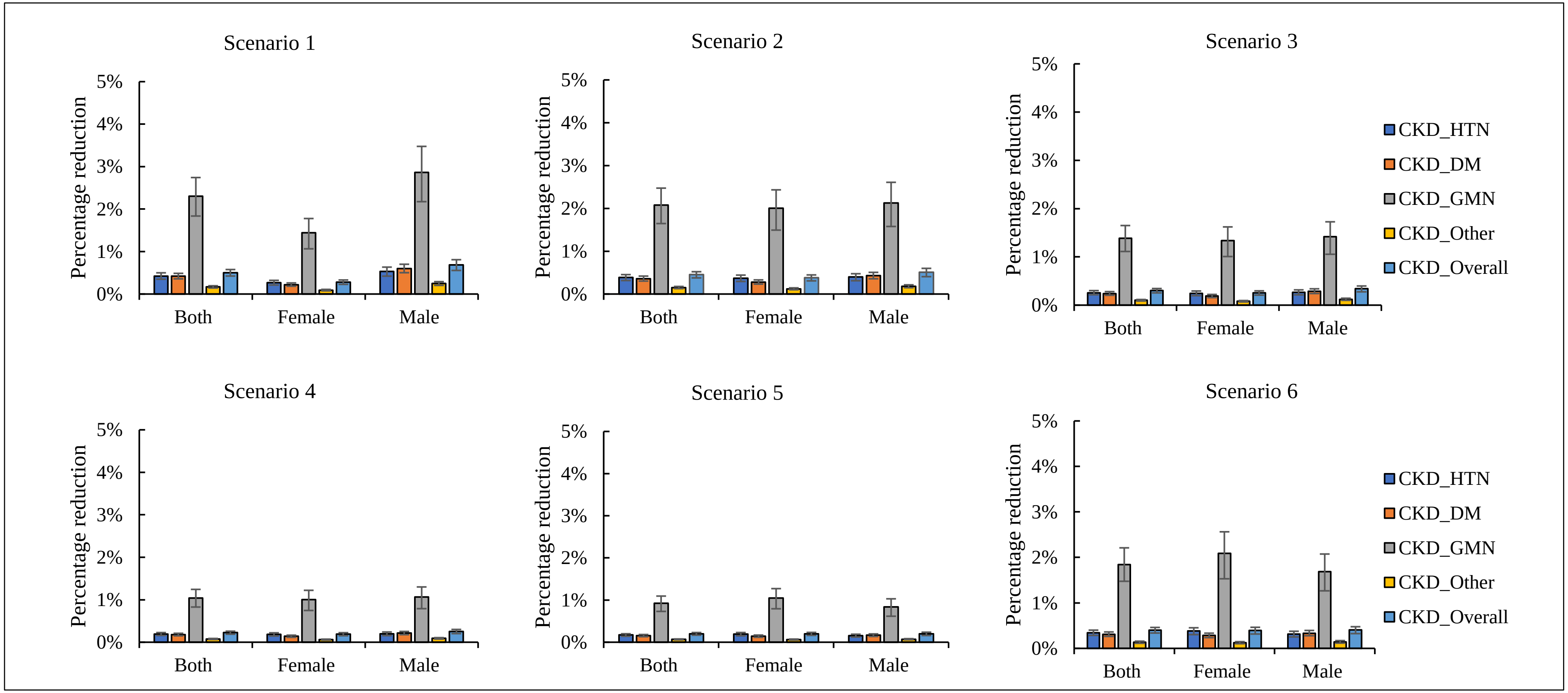

Figure S7: Sex-specific relative reduction in mortality from CKD by cause for all six scenarios between 2019 to 2030
Scenario 1: Reducing current salt intake to an average of 5 g/day (Australian Suggested Dietary Target), Scenario 2: 30% relative reduction in current salt intake (National Preventive Health Strategy, 2021-2030 target), Scenario 3: 20% relative reduction in current salt intake, Scenario 4: 15% relative reduction in current salt intake; Scenario 5: reduction in current salt intake by 1g; Scenario 6: reduction in current salt intake by 2g. Error bars reflect the 95% uncertainty intervals.
Scen, Scenario; CKD_HTN, chronic kidney disease due to hypertension; CKD_DM, chronic kidney disease due to diabetes mellitus; CKD_GMN, chronic kidney disease due to glomerulonephritis; CKD_Other, chronic kidney disease due to other or unspecified causes.

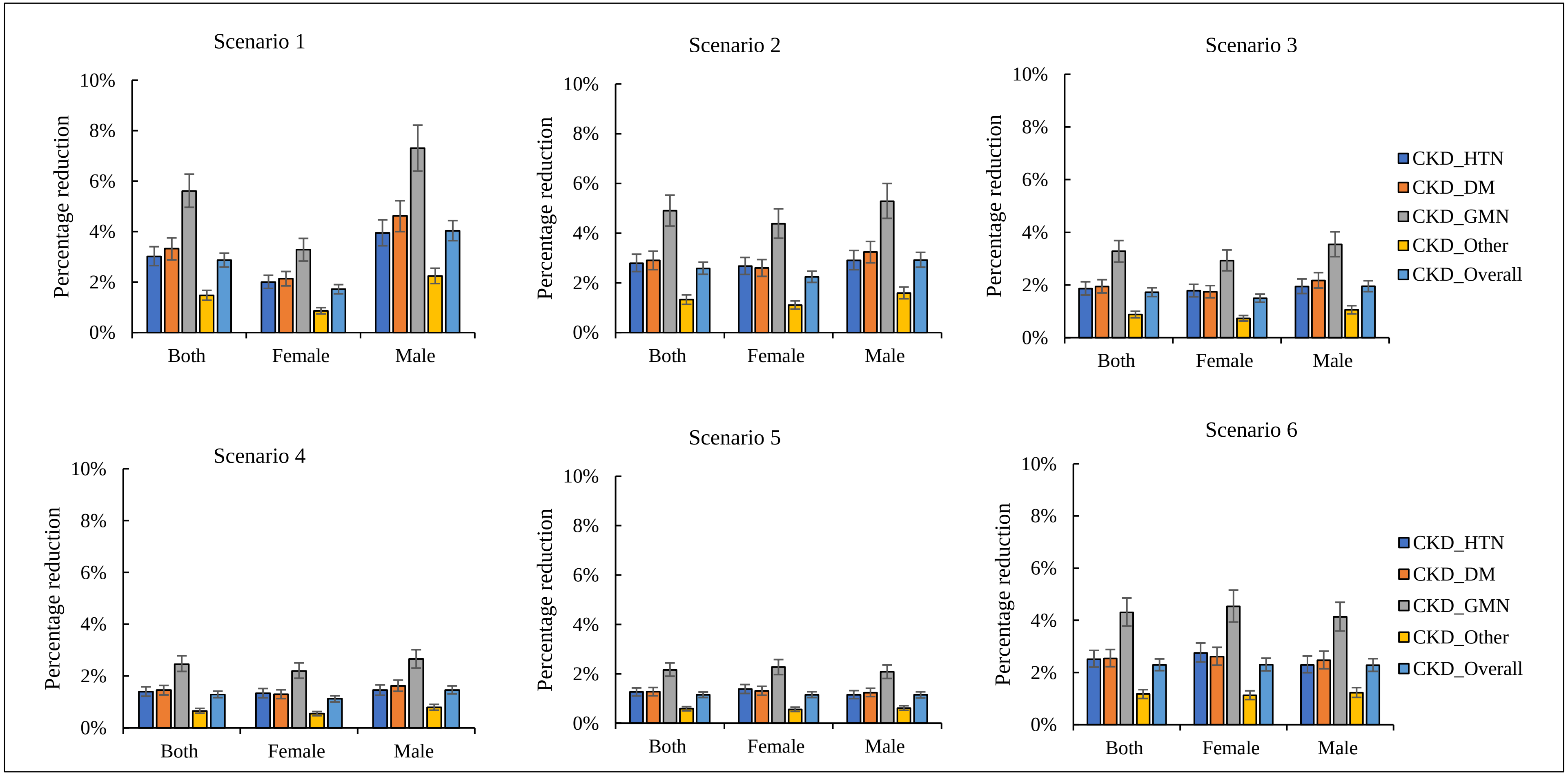

Figure S8: Lifetime sex-specific relative reduction in mortality from CKD by cause for all six scenarios

Scenario 1: Reducing current salt intake to an average of 5 g/day (Australian Suggested Dietary Target), Scenario 2: 30% relative reduction in current salt intake (National Preventive Health Strategy, 2021-2030 target), Scenario 3: 20% relative reduction in current salt intake, Scenario 4: 15% relative reduction in current salt intake; Scenario 5: reduction in current salt intake by 1g; Scenario 6: reduction in current salt intake by 2g. Error bars reflect the 95% uncertainty intervals.

Scen, Scenario; CKD_HTN, chronic kidney disease due to hypertension; CKD_DM, chronic kidney disease due to diabetes mellitus; CKD_GMN, chronic kidney disease due to glomerulonephritis; CKD_Other, chronic kidney disease due to other or unspecified causes.

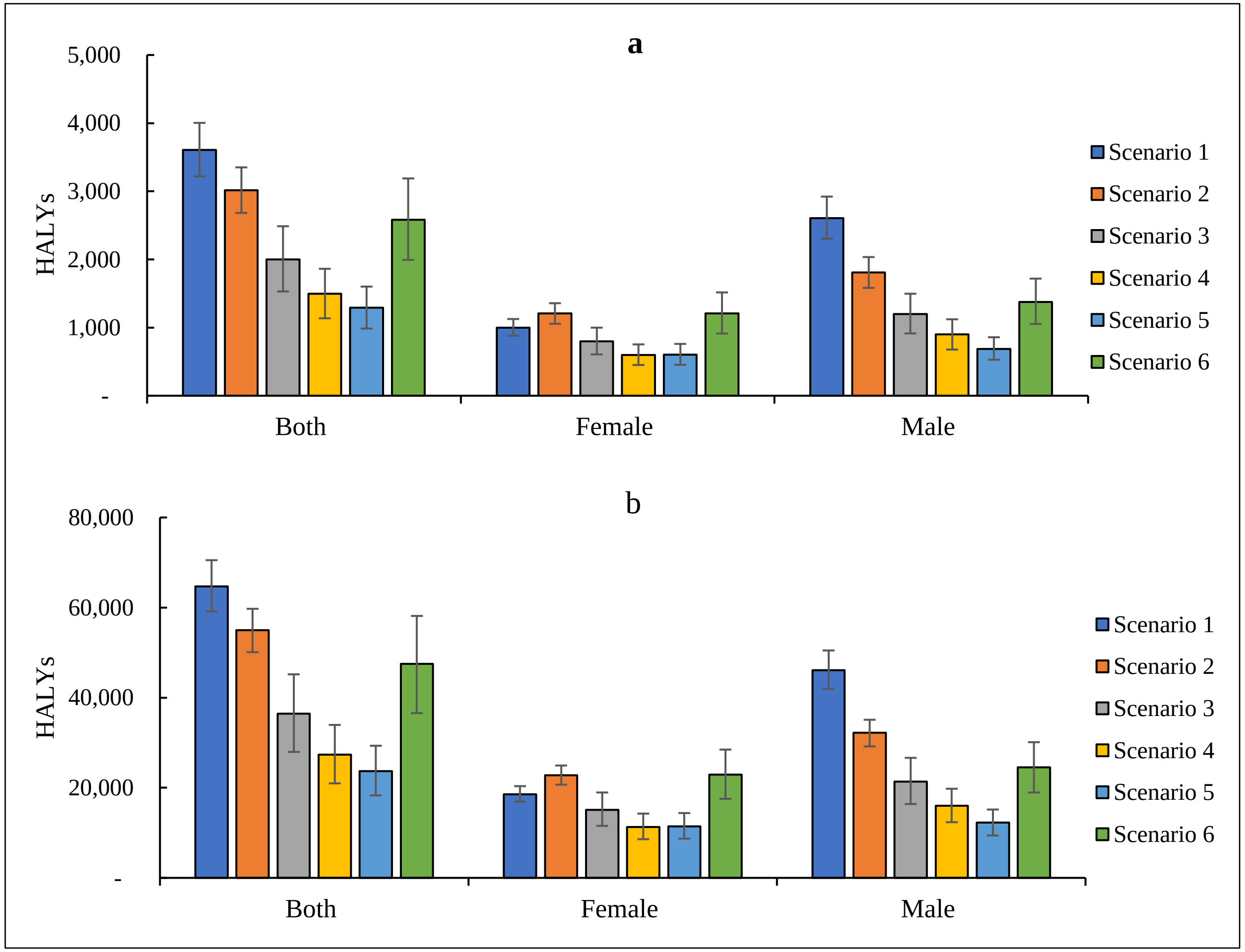

Figure S9: Overall and sex-specific HALYs (discounted at 3%) gained between 2019 to 2030 (panel A) and over the lifetime (panel B) of the 2019 adult population
Scenario 1: Reducing current salt intake to an average of 5 g/day (Australian Suggested Dietary Target), Scenario 2: 30% relative reduction in current salt intake (National Preventive Health Strategy, 2021-2030 target), Scenario 3: 20% relative reduction in current salt intake, Scenario 4: 15% relative reduction in current salt intake; Scenario 5: reduction in current salt intake by 1g; Scenario 6: reduction in current salt intake by 2g; HALY, health-adjusted life years. Error bars reflect the 95% uncertainty intervals.

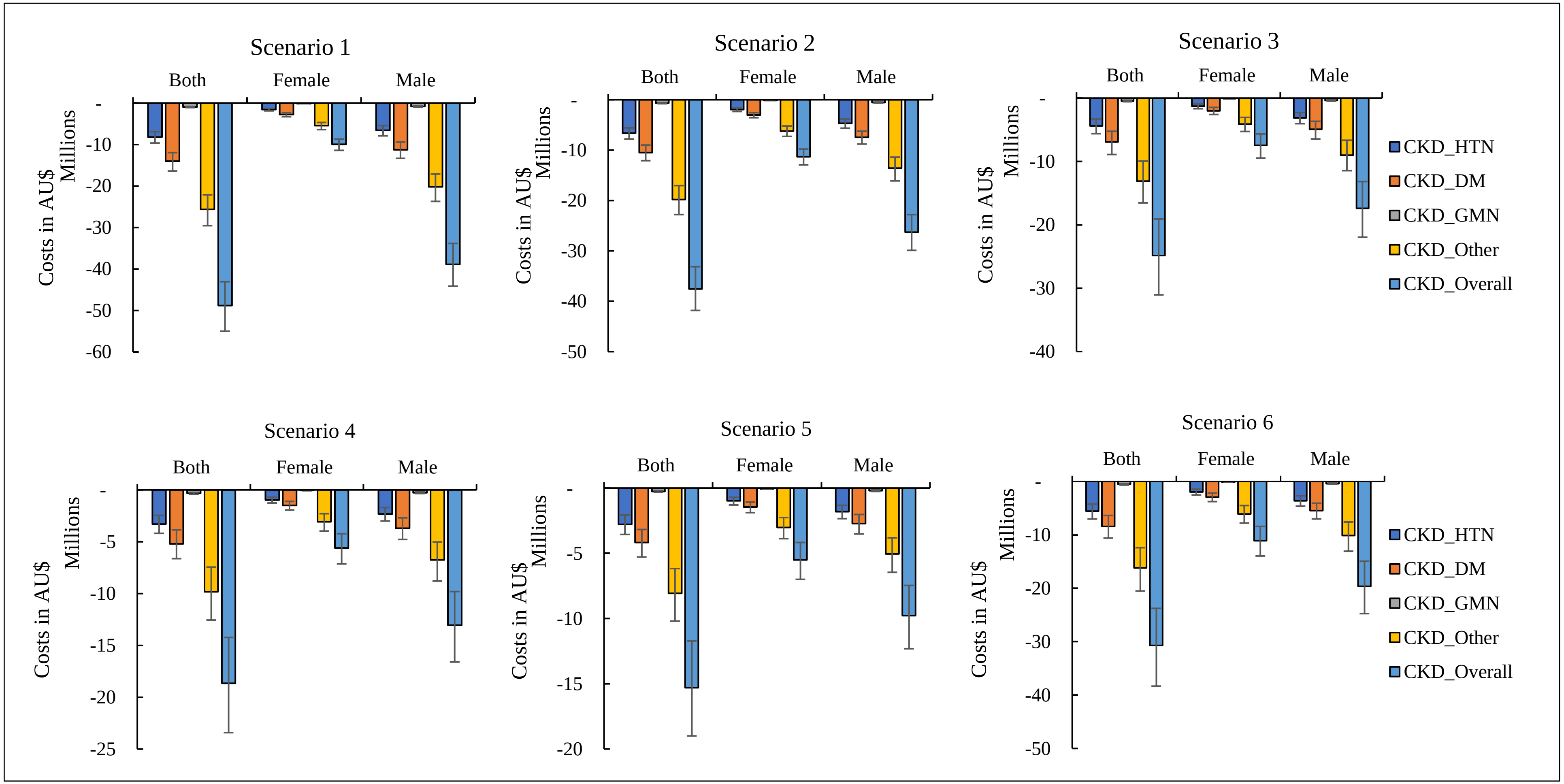

Figure S10: Healthcare costs savings (discounted at 5%) between 2019 to 2030 by CKD cause and gender

Scenario 1: Reducing current salt intake to an average of 5 g/day (Australian Suggested Dietary Target), Scenario 2: 30% relative reduction in current salt intake (National Preventive Health Strategy, 2021-2030 target), Scenario 3: 20% relative reduction in current salt intake, Scenario 4: 15% relative reduction in current salt intake; Scenario 5: reduction in current salt intake by 1g; Scenario 6: reduction in current salt intake by 2g. Error bars reflect the 95% uncertainty intervals.

Scen, Scenario; CKD_HTN, chronic kidney disease due to hypertension; CKD_DM, chronic kidney disease due to diabetes mellitus; CKD_GMN, chronic kidney disease due to glomerulonephritis; CKD_Other, chronic kidney disease due to other or unspecified causes; AU$, 2019 Australian Dollars.

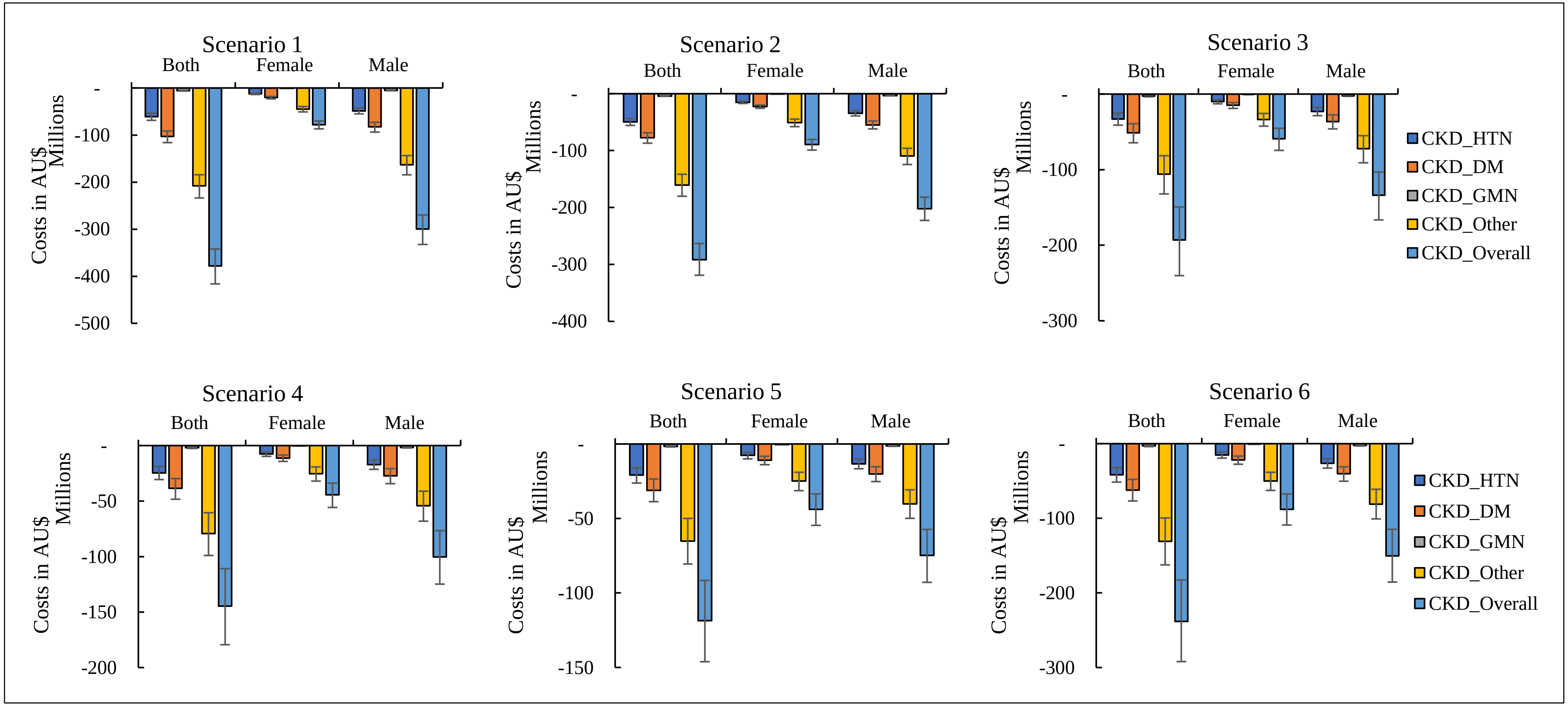

Figure S11: Healthcare costs savings (discounted at 5%) by CKD cause and gender over the lifetime of the 2019 adult population
Scenario 1: Reducing current salt intake to an average of 5 g/day (Australian Suggested Dietary Target), Scenario 2: 30% relative reduction in current salt intake (National Preventive Health Strategy, 2021-2030 target), Scenario 3: 20% relative reduction in current salt intake, Scenario 4: 15% relative reduction in current salt intake; Scenario 5: reduction in current salt intake by 1g; Scenario 6: reduction in current salt intake by 2g. Error bars reflect the 95% uncertainty intervals.
Scen, Scenario; CKD_HTN, chronic kidney disease due to hypertension; CKD_DM, chronic kidney disease due to diabetes mellitus; CKD_GMN, chronic kidney disease due to glomerulonephritis; CKD_Other, chronic kidney disease due to other or unspecified causes; AU$, 2019 Australian Dollars.
